# Improved Prediction of Bacterial Genotype-Phenotype Associations Using Interpretable Pangenome-Spanning Regressions

**DOI:** 10.1128/mBio.01344-20

**Published:** 2020-07-07

**Authors:** John A. Lees, T. Tien Mai, Marco Galardini, Nicole E. Wheeler, Samuel T. Horsfield, Julian Parkhill, Jukka Corander

**Affiliations:** a MRC Centre for Global Infectious Disease Analysis, Department of Infectious Disease Epidemiology, Imperial College London, London, United Kingdom; b Oslo Centre for Biostatistics and Epidemiology, Department of Biostatistics, University of Oslo, Oslo, Norway; c Biological Design Center, Boston University, Boston, Massachusetts, USA; d Centre for Genomic Pathogen Surveillance, Wellcome Sanger Institute, Wellcome Genome Campus, Hinxton, United Kingdom; e Department of Veterinary Medicine, University of Cambridge, Cambridge, United Kingdom; f Helsinki Institute of Information Technology, Department of Mathematics and Statistics, University of Helsinki, Helsinki, Finland; University of Maryland School of Medicine

**Keywords:** elastic net, pangenome, phenotype prediction

## Abstract

Discovery of genetic variants underlying bacterial phenotypes and the prediction of phenotypes such as antibiotic resistance are fundamental tasks in bacterial genomics. Genome-wide association study (GWAS) methods have been applied to study these relations, but the plastic nature of bacterial genomes and the clonal structure of bacterial populations creates challenges. We introduce an alignment-free method which finds sets of loci associated with bacterial phenotypes, quantifies the total effect of genetics on the phenotype, and allows accurate phenotype prediction, all within a single computationally scalable joint modeling framework. Genetic variants covering the entire pangenome are compactly represented by extended DNA sequence words known as unitigs, and model fitting is achieved using elastic net penalization, an extension of standard multiple regression. Using an extensive set of state-of-the-art bacterial population genomic data sets, we demonstrate that our approach performs accurate phenotype prediction, comparable to popular machine learning methods, while retaining both interpretability and computational efficiency. Compared to those of previous approaches, which test each genotype-phenotype association separately for each variant and apply a significance threshold, the variants selected by our joint modeling approach overlap substantially.

## Abstract (precis)

Being able to identify the genetic variants responsible for specific bacterial phenotypes has been the goal of bacterial genetics since its inception and is fundamental to our current level of understanding of bacteria. This identification has been based primarily on painstaking experimentation, but the availability of large data sets of whole genomes with associated phenotype metadata promises to revolutionize this approach, not least for important clinical phenotypes that are not amenable to laboratory analysis. These models of phenotype-genotype association can in the future be used for rapid prediction of clinically important phenotypes such as antibiotic resistance and virulence by rapid-turnaround or point-of-care tests. However, despite much effort being put into adapting genome-wide association study (GWAS) approaches to cope with bacterium-specific problems, such as strong population structure and horizontal gene exchange, current approaches are not yet optimal. We describe a method that advances methodology for both association and generation of portable prediction models.

## INTRODUCTION

Bacterial genomics has recently entered an era of “big data.” Single cohorts with 10^4^ to 10^5^ samples, 10^8^ genetic variants, and corresponding extensive high-quality metadata are now publicly available (1, 2). In the context of bacterial populations, the challenge is to take large data sets consisting of whole genomes and metadata such as measured antimicrobial resistance or host disease status and identify genetic variants associated with antimicrobial resistance, host specificity, or virulence phenotypes. With enough independent observations, hypothesis-free machine learning methods can generate models which predict the phenotype of new isolates and potentially tell us something about the underlying genetic mechanisms. A deluge of recent papers have applied general predictive models to such data sets and have mostly showed high accuracy (3–8). However, some commentaries have been more cautious in their conclusions (9, 10).

The overall problem of relating microbial genotype to phenotype has generally been approached by genome-wide association study (GWAS) methods (11–13). Such methods determine whether there is sufficient evidence to conclude that a specific variant explains some proportion of the variation of a trait, after accounting for as many statistical artifacts as possible. Generally, these methods start by taking the following approach: consider the association between a phenotype and a single variant, evaluate this association while accounting for known covariates, including population structure, and then “scan” this test along the whole genome one variant at a time. Ideally, associated sets of variants will be investigated further to find causally associated variants within these sets of linked loci (known as fine-mapping), but even in the best case, this is extremely challenging (14, 15). It is particularly crucial for microbial GWAS methods to incorporate a correction for population structure in each test, the specifics of which vary between methods and data sets. Further adjustment may be necessary if confounders are genetically stratified due to sampling strategy. Despite the required adjustments, the simplicity of this method is one of its great strengths: it is quick and easy to apply, understand, and visualize. Useful extensions which allow the estimation of heritability (16), the proportion of phenotypic variance explained by genotype, and prediction of phenotype (by forming linear predictors from significant variants [17]) are also relatively simple to implement.

Large cohorts of bacterial sequences have also been a tempting target for machine learning and “deep learning” methods such as convolutional neural networks, which are able to relate arbitrary high-dimensional inputs to measured outputs with high accuracy and without the need for specialized model descriptions for each new problem (18). Rather than assessing evidence for association of individual variants as in GWAS, these methods instead aim to find a set of variants and a mapping to predict a trait as accurately as possible. They are potentially broadly applicable to any problem with vast amounts of data, though they perform best when the number of data points exceeds the number of dimensions. Unsurprisingly their uptake in sequence analysis (19, 20) and bacterial genomics specifically has been rapid (4, 5, 21). In general, the predictive variants in the identified sets may not fully overlap those significant by themselves, and the mapping does not necessarily lend itself readily to the same interpretation as *P* values in a GWAS.

However, some issues crucial to understanding bacterial populations remain unaddressed. First, bacterial populations tend to exhibit a strong population structure, meaning samples cannot be treated as independent. In the context of prediction, this can result in the selection of features unrelated to the phenotype but common to the background of associated strains (lineage effects). While not necessarily a problem in the training data set, if new data are drawn from different strains, this can lead to much poorer prediction than expected. Examples of this effect are ubiquitous in artificial intelligence, for example, automatically designed clocks which only work in the lab they were built (22, 23). In human genetics, a similar problem has arisen due to an overrepresentation of samples of European ancestry in genotype databases. This has led to polygenic risk scores, which were originally thought to be highly accurate predictors of disease liability, to have significantly lower accuracy in non-European ancestry samples, which make up most of the global population (24). Additionally, these methods are unable to deal with missing input data. Variant calling, either by read pileup to generate single nucleotide polymorphisms (SNPs) or from a graph, when conducted separately for each population, is likely to produce disparate input sets, and with very different minor allele frequencies. Without using a method which produces consistent variant calls in test data sets, the accuracy of predictive models is likely to be heavily overstated. As well as methodological approaches, more representative sampling of the pathogen population which does not oversample clonal lineages may be advisable.

Here, we set out to develop a method which combines the desirable attributes of both of these classes of approaches when analyzing the genetics underlying bacterial traits. We wished to retain the simplicity and interpretability of traditional GWAS approaches and combine this with the flexibility and accuracy of machine learning methods which can be fitted to the entire data set at once. These models were previously applied to human GWAS data sets for inference (25–27) and prediction (28).

This pangenome-wide approach reflects the polygenic nature of complex traits better than older fixed effect methods which must select only some population structure covariates to include (29). Unlike marginal tests (the standard single predictor test in GWAS), a genome-wide regression approach gives rise to an increase of resolution when sample size increases, as was previously noted in human GWAS (30–32). Additionally, simultaneously analyzing predictors together in a regression model means that interactions and correlation between the predictors (e.g., population structure) may be included implicitly (33).

Using large genomic data sets from four different species and sixteen varied phenotypes, we find that an elastic net model (33) selects similar variants to a GWAS and does not sacrifice its major advantage of quantitative model interpretability. Using simulated data, we demonstrate improved power, but an increase in false-positive rate, compared to that of linear mixed models. We illustrate this use in practice on antibiotic resistance phenotypes in two species and show further results which find similar accuracy between new machine learning and simpler approaches, consistent with previous studies (4, 5, 34).

Our approach models the entire pangenome of the population to include the large proportion of variation which resides in the accessory genome. It explicitly addresses issues of population structure and consistent performance between trained and new (test) data sets. The method is broadly usable, not requiring programming knowledge or manual adjustments for new data sets, and allows for the sharing of models between researchers. We have implemented the elastic net model in the pyseer microbial package as a new “prediction” module and consistent pangenome variant calling in two further packages. An extensive tutorial for all of these methods is available online (https://pyseer.readthedocs.io/en/master/predict.html).

## RESULTS

### Method overview.

The elastic net uses a linear prediction model as with a standard linear regression run between a phenotype and all genetic variants and tries to find slopes (effect sizes) for all the variants which best predict the phenotype. A shrinkage term (λ) is used to prevent overfitting, adding a cost to each fitted slope proportional to its value. This has the effect of making many of the genetic variants have a slope of zero, and so they can be removed from the model entirely. To predict the phenotype in new samples, these fitted slopes form a simple linear model that can be applied to a new set of input variants. Furthermore, this also allows the calculation of *R*^2^, the variance in phenotype due to the variance of all genetic effects—the heritability (total effect of genetics) of the phenotype.

We use an alignment-free representation variation as input. Alignment-free approaches have proven particularly popular in bacterial populations, removing the need for selection of a particular reference and simultaneously modeling both gene content and sequence variation (11, 12, 35). Many methods have previously used k-mers, which are short sequence words of length k. The DBGWAS method proposes connecting the overlaps of these k-mers in a compressed de Bruijn graph (DBG) so that k-mers are extended using adjacent sequence information in the population, forming unitigs present in exactly the same set of samples as their constituent k-mers (36). We followed the same approach here.

As the elastic net includes predictors that are correlated due to population structure in the same model fitting, it will typically downweight these, to some extent incorporating this evolutionary history into its model. However, as we note below, effect size alone does not have the same interpretation as a *P* value from GWAS and must be considered along with minor allele frequency (MAF) and other confounders. To include a population structure explicitly in the model, we first divide the population into strains (or lineages). Each sample’s contribution to the fitting is then downweighted by the prevalence of the strain in the elastic net so that repeated observations of the same genotype count for less, known as “sequence reweighting.” We also use this to select the value shrinkage term λ. We designed a “leave-one-strain-out” (LOSO) cross-validation rather than randomly leaving samples out. This aims to avoid correctly predicting the strain (which is frequently correlated with phenotype) rather than the phenotype itself, since such an approach is less robust when common strains dominate the data or when the fitted population model is not representative of a population in which predictions will be made. When applied together, we refer to this as a “weighted” model, as opposed to “unweighted” models which use neither of these adjustments.

### Prediction within and between cohorts without sacrificing model interpretability.

Whole-genome models can be used to construct a linear model to predict phenotypes in new data. In this section, we evaluate these predictions compared to those of other models and variant calling methods using a variety of data sets and phenotypes.

**(i) Mycobacterium tuberculosis resistance is equally well predicted by the elastic net.** We first evaluated the predictive performance of our models, with and without population structure control, compared to that of a more complex deep learning model. We used an M. tuberculosis data set with antibiotic resistance to four first-line antibiotics (rifampin, isoniazid, ethambutol, and pyrazinamide). As M. tuberculosis has no accessory genome and minimal core gene variation (37), comparison with more complex models and a SNP alignment is possible. Previous work has evaluated the use of a multitask deep neural network and, when comparing this to lasso regression, found comparable accuracy (5). Using the same input of ∼6,500 SNPs and short insertion/deletions across the allele-frequency spectrum for 3,566 samples (split into training and test data sets) led to average false-negative rates of 2% ± 3% in the unweighted model and 3% ± 4% in the weighted model and false-positive rates of 11% ± 8% in the unweighted model and 12% ± 10% in the weighted mode. The elastic net therefore gives similar performance to the lasso as well as the more complex neural network (see Table S1 in the supplemental material), as was also shown by the original study authors (5). It is, however, much easier and faster to run on standard hardware (run time of <1s on a central processing unit [CPU] versus ∼3 min on a high-end graphics processing unit [GPU]) and gives results which are far more readily interpretable.

10.1128/mBio.01344-20.7 TABLE S1 Prediction accuracy on the Mycobacterium tuberculosis dataset. For resistance to each of the four front-line treatments, we fitted a model with and without sequence reweighting and compared accuracy on a uniform random test/training split. The results from the neural network in Chen et al. (5) (wide and deep neural network [WDNN]) are also included. TP, true positives; TN, true negatives; FP, false positives; FN, false negatives. The number of samples in each lineage is as follows: L1, 452; L2, 448; L3, 207; L4, 73. Download Table S1, PDF file, 0.1 MB. Copyright © 2020 Lees et al. 2020 Lees et al. This content is distributed under the terms of the Creative Commons Attribution 4.0 International license.

In this case, the weighted model generally performs slightly worse than an unweighted model. The population structure of this sample is relatively simple, with four distinct lineages. This is likely well captured implicitly by the unweighted model, and so the categorical weighting is of lower resolution. Sequence reweighting is instead expected to be more effective in data sets with more complex structures or when adding in samples which are genetically distant from the training set (38, 39), which we explore further in the next section. Applying these weights allowed us to easily see that the majority of errors occurred in lineage I, which has deep branches forming genetically separated subclades, with generally perfect prediction in the other three lineages.

**(ii) Prediction of pneumococcal resistance using different variant types.** We also investigated the advantages of the use of unitigs over other variant calling methods. Using the same Streptococcus pneumoniae antimicrobial resistance in children (SPARC) data set described above for β-lactam and erythromycin resistance, we compared computational resources and prediction accuracy using SNPs, k-mers, and unitigs (Table 1).

**TABLE 1:** Predicting antibiotic resistance in the SPARC collection using different variant types^*a*^

Variant type	Phenotype	No. selected	FPR (%)	FNR (%)	CPU time (min)	Memory usage (Gb)
SNPs (90,000), 3.6 Mb on disk	β-Lactam	4,374	3	7	4.4	1.3
	Erythromycin	2,341	3	63	4.1	1.3
Unitigs (730,000), 25 Mb on disk	β-Lactam	8,247	5	7	49.7	18
	Erythromycin	1,591	9	39	52.6	6.9
k-mers (10 million), 603 Mb on disk	β-Lactam	15,121	6	7	420	212

a Using a training/test split of 2:1, prediction accuracy of two phenotypes was tested using 90,000 SNP calls from mapping to a reference genome, and with 730,000 unitigs. We also tested prediction using 10 million variable-length k-mers to illustrate the heavy computational resource use in even a relatively small data set. File sizes are for the sparse data structures we employ.

We found that for β-lactam resistance, all three variant types gave similar predictive accuracy, with the elastic net able to select a small proportion of the total input variants in each case and apparently fairly insensitive to the far greater noise present in the higher dimensional variant types. As this resistance is due to allelic variation in core genes, we expect all three types to tag the causal variation equally well. For erythromycin, where causal variants are not all found in core genes, we observed a reduction in the false-negative rate when using unitigs. Computational usage increased roughly as *NM* (*N*, number of samples; *M*, number of variants). For common variants, *M* reaches an asymptote for a given population: the main requirement is therefore based on *N*. For all methods, the CPU time was modest, but memory usage may pose a problem. SNPs are tractable on a laptop, but unitig analysis likely requires a computing cluster for the model fitting (using a fitted model on test data requires negligible resources). k-mers require an enormous amount of memory, which would not scale to larger data sets. Though the unitig analysis was easy to schedule on our cluster, future improvements to reduce memory use could include accessing the variants as they are needed from a disk or fitting the elastic net in chunks, with resampling (40, 41).

**(iii) Reduced intercohort accuracy is ameliorated with consistent genetic calls and population structure control.** Random splits of single data sets in test and training data, while convenient for analysis, may mask inter-data set differences such as class imbalance (different resistance rates), unobserved lineages, and technical errors (variant calling) (10). To test a more realistic example, where a previously fitted model is used to predict resistance status in new unobserved data, we set up a prediction experiment using genomic data from three large very different pneumococcal cohorts with β-lactam resistance: SPARC (603 U.S. children covering introduction of vaccine); Maela (3,162 unvaccinated infants and mothers); global pneumococcal sequence (GPS) (5,820 globally distributed samples, mostly vaccinated). We counted unitigs for each population and used these to train a predictive model. These models were evaluated on the data they were trained on and on the other two cohorts by using consistently named unitigs from unitig-caller (Table 2). The resources used were as follows: SPARC, 5 Gb random access memory (RAM), 0.6 h; Maela, 30 Gb RAM, 2.5 h; GPS, 3.1 h. The majority (∼80%) of the CPU time used was for reading variants from text files, making subsequent fitting faster. The distributions of unitig sizes are shown in Fig. S1.

**TABLE 2:** Comparison of intra- and intercohort prediction accuracy^*a*^

Model	No. of selected unitigs (% in *pbp* genes)	Accuracy^*b*^								
		SPARC data			Maela data			GPS data		
		FNR	FPR	*R*^2^	FNR	FPR	*R*^2^	FNR	FPR	*R*^2^
Sequence reweighting										
SPARC	5,251 (10)	0.063	0.024	0.837	0.007	0.239	0.439	0.149	0.134	0.505
Maela	6,645 (14)	0.446	0.005	0.276	0.082	0.042	0.760	0.029	0.382	0.425
GPS	894 (4)	0.011	0.411	0.447	0.144	0.177	0.458	0.094	0.200	0.545
Without weighting										
SPARC	7,261 (10)	0.040	0.013	0.901	0.012	0.163	0.487	0.165	0.130	0.487
Maela	8,705 (9)	0.397	0.011	0.339	0.063	0.036	0.805	0.049	0.322	0.449
GPS	7,511 (2)	0.050	0.152	0.656	0.319	0.026	0.452	0.129	0.037	0.864

a For each prediction, the error rates are listed along with overall *R*^2^. For SPARC and Maela, phenotype was binary (resistant/sensitive); for GPS, phenotype was continuous (MIC). Where conversion was needed, we applied the standard breakpoint of MIC > 0.12 mg/liter for resistance.

b Shaded cells are within-cohort. FNR, false-negative rate; FPR, false-positive rate.

10.1128/mBio.01344-20.1 FIG S1 Histograms of the unitig length distributions in S. pneumoniae data sets. Minimum unitig size is by definition the de Bruijn graph k-mer size, which was chosen to be 31 for all datasets. Download FIG S1, PDF file, 0.4 MB. Copyright © 2020 Lees et al. 2020 Lees et al. This content is distributed under the terms of the Creative Commons Attribution 4.0 International license.

Between-cohort predictive accuracy was considerably lower than within-cohort accuracy but still outperformed an intercept-only model in all cases. The use of unitigs proved successful: repeating the SPARC-Maela comparisons with SNPs led to extremely poor predictions for every sample, as the selected SNPs were called as missing in the other cohort, leading to a mean value prediction for every sample (true negatives, 1,661; false positives, 0; false negatives, 1,282; true positives, 0). To fix this issue with SNPs would likely require a labor-intensive mapping and joint recalling of variation, whereas with sequence elements, the simple search implemented in unitig-caller can be used. To deal with missing unitig data, which may truly be missing or miscalled, we assumed it was all truly missing. On the same SPARC-Maela comparison (false-negative rate [FNR], 0.007; false-positive rate [FPR], 0.239; *R*^2^, 0.439), this gave very similar results to those using mean allele frequency [AF] imputation for missing unitigs (FNR, 0.008; FPR, 0.232; *R*^2^, 0.453).

Depending on the specific model and data set combination, errors can much more commonly be type I or type II, possibly reflecting class imbalance, despite overall resistance rates in the pneumococcus being stable (42). The GPS cohort gave the worst performing model, despite it being the largest collection. This is a very genetically diverse sample, which introduces more potential for confounding lineage effects to enter the model. Furthermore, this cohort is a mix of sequences isolated from cases of asymptomatic carriage and disease, whereas SPARC and Maela contain only asymptomatic carriage cases. The GPS cohort is enriched with more-virulent strains, which have more frequently faced treatment with antibiotics and have a higher rate of resistance (1, 43).

We also note that the area under the curve (AUC) of the receiver operating characteristic (ROC) is misleadingly high (0.9185/0.9728 for the weighted/unweighted GPS model) and would encourage the reporting of error rates as more intuitive summaries of accuracy for bacterial traits such as resistance.

We found that sequence reweighting generally reduced prediction accuracy for this phenotype, although it is the LOSO strategy in particular which gave slightly more representative accuracy estimates for out-of-cohort prediction (when comparing within data set with between data set prediction, *R*^2^ was 72% higher with sequence reweighting versus 82% higher without sequence reweighting), and more of the selected variants were in the causal loci.

**(iv) Virulence phenotypes can be predicted with sequence reweighting, preventing overestimation of accuracy.** Most work on prediction of bacterial phenotypes has focused on antibiotic resistance, but many more complex phenotypes relating to bacterial virulence are now available. For these phenotypes, which are under weaker or no selection, instead of a few strong effects, multiple smaller effects are expected in the genome (44–46). Therefore, a model which may include more of these effects, which would be missed with a *P* value threshold, may be expected to perform well.

**FIG 1:**
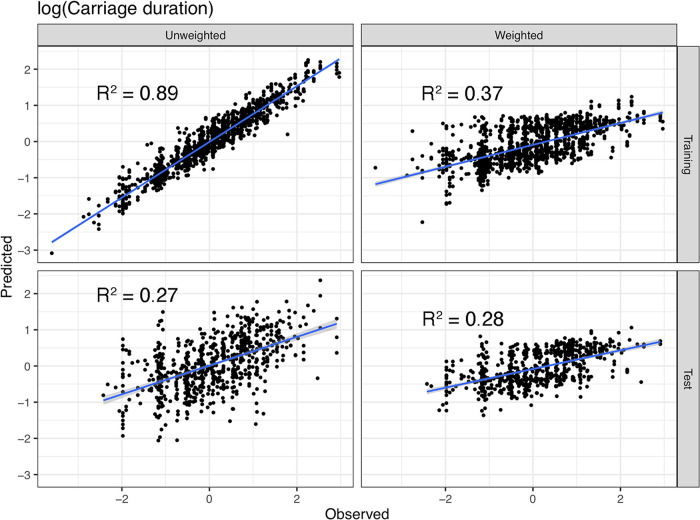
Prediction of carriage duration and the effect of sequence reweighting on heritability estimation. For the same training/test split, each panel shows observed log(carriage duration) values on the *x* axis and model-predicted values on the *y* axis, with a fitted linear regression. (Left) Unweighted model on the training data (top) and test (bottom). (Right) The same for the model with sequence reweighting.

We applied our method to predict the duration of asymptomatic carriage in a subset of the Maela cohort, which can easily be visualized in the manner of a linear regression (Fig. 1). We show the observed versus predicted values for the training and test sets, both with and without sequence reweighting. In the unweighted training set, *R*^2^ (and heritability [*h*^2^]) was 0.89 (Fig. 1, top left), but the test *R*^2^ was only 0.27 (Fig. 1, bottom left), showing clear overfitting. With sequence reweighting and LOSO, the training and test estimates were much closer (0.37 and 0.28, respectively) (Fig. 1, right). In this case, sequence reweighting gave a more realistic heritability estimate. *h*^2^ was previously estimated to be 0.634 using phylogenetic pairs, and 0.445 using restricted maximum likelihood (REML)—these may be overestimates, especially as the revised estimates introduced here used more information from the genome.

We also tested virulence prediction in two streptococcal species, which would be a useful application for routine pathogen surveillance but has not been as thoroughly explored as resistance prediction. Using Streptococcus pneumoniae isolated from Dutch adults, we fitted a model which selected 9,701 unitigs. This model was able to predict meningitis versus carriage genomes (test FPR, 0.059; FNR, 0.12) and gave a similar *h*^2^ estimate to that originally reported (0.65 versus 0.70). Comparing tissue infection with carriage of Streptococcus pyogenes gave a model with 5,817 unitigs, which had a higher error rate than the S. pneumoniae model (test FPR, 0.24; FNR, 0.25), but the phenotype also had a correspondingly lower *h*^2^ estimate of 0.343. Detailed performance of these models is given in Table S2.

10.1128/mBio.01344-20.8 TABLE S2 Prediction accuracy of the elastic net in other binary datasets, all of which are S. pneumoniae. Download Table S2, PDF file, 0.1 MB. Copyright © 2020 Lees et al. 2020 Lees et al. This content is distributed under the terms of the Creative Commons Attribution 4.0 International license.

### Power and false-discovery rate compared to GWAS using simulated phenotypes.

To test the characteristics of the elastic net compared to GWAS approaches, we simulated phenotype data from the Maela population (3,162 S. pneumoniae genomes) (Table 3) using previously defined SNP variation (47). We chose either 5, 25, 100, or 300 true causal variants with an effect size of 4 (similar to a penetrant antimicrobial resistance variant) either

- chosen uniformly at random across the genome, after linkage disequilibrium (LD) pruning (no variants with *R*^2^ > 0.9).
- chosen uniformly at random from 1 to 3 prespecified genes (*pbpX-pbp2x*, *penA-pbp2b*, and *pbp1a*).

**TABLE 3:** Summary of data sets tested^*a*^

Data set name	Species	Phenotype(s) and split	Reference	No. of samples	No. of samples for training/test	No. of genetic features
TB	Mycobacterium tuberculosis	First-line antibiotic resistance: rifampicin, 1,285:2,257; isoniazid, 1,553:2,011; pyrazinamide, 702: 2,445; ethambutol, 975:2,551	5	3,566	2,377/1,189	6,400 (SNPs)
N. gonorrhoeae	Neisseria gonorrhoeae	Antibiotic resistance MICs: azithromycin, cefixime, ciprofloxacin, penicillin, and tetracycline	53, 61, 83, 84	1,595	NU^*b*^	550,000 (unitigs)
GAS	Streptococcus pyogenes	Virulence, 1,093:637	46	1,730	1,154/576	1.1 million (unitigs)
SPARC	Streptococcus pneumoniae	Antibiotic resistance MICs: penicillin, erythromycin	47, 85	603	400/203	90,000 (SNPs), 730,000 (unitigs), 10 million (k-mers)
Maela	Streptococcus pneumoniae	Carriage duration; antibiotic resistance: penicillin, 1,661:1,282; erythromycin, 802:2,355; trimethoprim, 609:2,548	12, 44	3,162 (antibiotic resistance), 2,017 (carriage duration)	1,404/703 (carriage duration)	121,000 (SNPs), 1.6 million (unitigs)
GPS	Streptococcus pneumoniae	Antibiotic resistance (penicillin)	1	5,820	NU	1.7 million (unitigs)
Netherlands	Streptococcus pneumoniae	Meningitis/carriage, 693:1,144	45	1,837	1,225/612	690,000 (unitigs)

a Each data set has a name by which it is referred to in the text. Most data sets have multiple phenotypes available, especially where multiple different antibiotic resistances are routinely phenotyped. Data sets without a training/test split were not evaluated for internal prediction ability as they were instead used with more stringent external validation data sets or were used for GWAS only, and all available samples were used to fit the model.

b NU, not used.

We chose the first setting to emulate a polygenic trait, with many variants of roughly equal effects associated across the genome. LD pruning was only used to select causal variants, and all variants were used as input to the model. The second setting more closely resembles antibiotic resistance, where multiple alleles in either one or a small number of genes contribute to the effect, with multiple occurrences independent of genetic background. We ran the elastic net (α = 0.01) and lasso regression (α = 1) as well as both GWAS models (fixed effects and linear mixed model) previously implemented in pyseer. Variants were output by the genome-wide model if they had a nonzero coefficient and by the GWAS models if their *P* value exceeded a significance threshold of 0.05 after Bonferroni correction. For each simulated data set and method, counting true positives (TP), true negatives (TN), false positives (FP), and false negatives (FN), we calculated the power, the proportion of true causal variants in the output: TP/(TP + FN). This allowed us to analyze the overlap of selected variables which gave good prediction with those found in a GWAS, which are individually associated with a phenotype of interest and may therefore provide mechanistic insight into the trait being analyzed. We also calculated the proportion of false positives in the output, the number of variants selected in the output which are not true causal variants divided by the total number of variants being tested: FP/(TP + FP + FN + TN). This is a measure of the “noise” in the selected predictor set.

First, our simulations were able to show that using the correlation filtering step (reducing input size by 25%) reduced power on average by 4% and 8% in the worst case (see Table S3), where many small-effect variants are spread across the genome and with no appreciable power loss with smaller or more concentrated causal variants. The sample correlation values are positively skewed due to population structure, and so filtering all variants with a sample correlation below the mean value rather than a quantile leads to an unacceptably high loss of power, as many causal variants would be removed. This quantile filter can therefore be used on large data sets to reduce CPU and memory usage with little effect on the variants selected, but if possible, the full set of variants should generally be modeled in its entirety.

10.1128/mBio.01344-20.9 TABLE S3 True causal variants retained after filtering on sample correlation. In parenthesis is the total number of the retained variants. (a) Varied sample sizes, uncorrelated true variants are chosen from across the genome. (b) Varied sample sizes, variants chosen from *pbpX*. (c) Varied sample sizes, variants from both regions. (d) Varied heritability, variants from both regions. Download Table S3, PDF file, 0.1 MB. Copyright © 2020 Lees et al. 2020 Lees et al. This content is distributed under the terms of the Creative Commons Attribution 4.0 International license.

**FIG 2:**
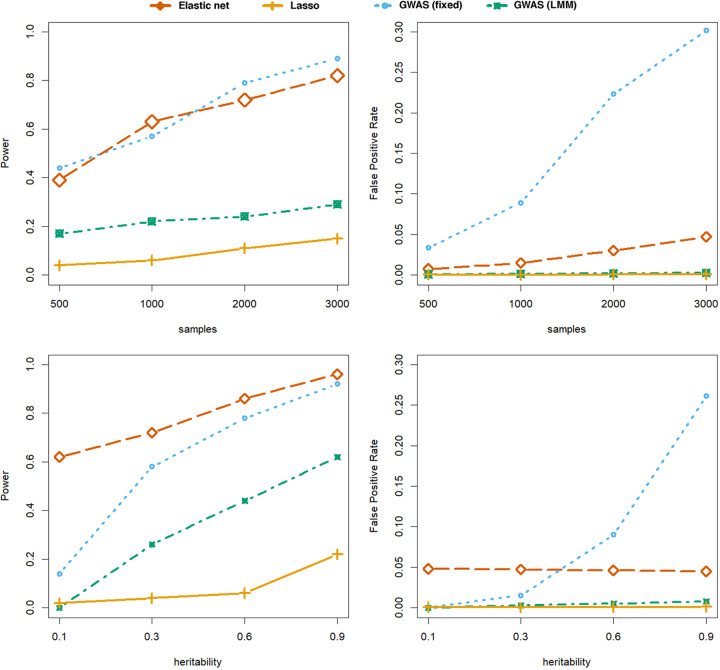
Power and false-positive rates in the simulation study set up to resemble antibiotic resistance genotype-phenotype architecture. (Top) The effect of sample size, with 100 causal variants in the *pbp2x* gene and a binary phenotype. (Bottom) The effect of phenotype heritability, with 50 causal variants spread across the three *pbp* genes and a continuous phenotype. Multivariate methods tested were the elastic net with default α (red) and Lasso regression (orange). Univariate methods were the fixed effects/seer model (blue) and the FastLMM linear mixed model (green).

Over all of our simulations, we found that either the elastic net or a fixed-effect GWAS had the highest power depending on the setting, and both always had higher power than the linear mixed model (Fig. 2 and Fig. S2). This is consistent with expectations from prior literature in multicellular organisms (25, 26, 40). The elastic net performed better in situations where the heritability was low or causal variants were spread out across the genome. This is expected for less penetrant traits such as carriage duration (44) and transmissibility (48). There was slightly lower power for all methods with binary phenotypes, and this decrease was more pronounced in the linear mixed model, possibly due to being the only model that used a Gaussian error structure in both settings.

10.1128/mBio.01344-20.2 FIG S2 Power and false-positive rates of GWAS and elastic net regression methods. (a) Different numbers of true variants were chosen from the *pbpX* gene, sample size was varied. (b) Different numbers of true variants were chosen from across the genome, sample size was varied. (c) Fifty true variants were chosen from different set ups: LD-pruned variants across the genome, and the genes *pbpX*, *pbp1a* and *penA*. Sample size was varied. (d) Fifty true variants were chosen from different set ups: LD-pruned variants across the genome, *pbpX*, *pbp1a*, and *penA*. Sample size was varied. (e) Varied heritability of a continuous phenotype. Fifty true variants were chosen from one or two genes. Sample size was fixed at 3,000. (f) Varied heritability of a binary phenotype. Fifty true variants were chosen from one, two, or three genes. Sample size was fixed at 3,000. Download FIG S2, PDF file, 1.0 MB. Copyright © 2020 Lees et al. 2020 Lees et al. This content is distributed under the terms of the Creative Commons Attribution 4.0 International license.

However, in exchange for reduced power, the linear mixed model consistently showed the best control of false positives in all settings, always <5%. In contrast, the fixed-effect model had a much greater false-positive rate than any other method, which grew both with sample size and heritability. The elastic net’s false-positive rate was typically <5% and was robust across the ranges of heritability tested, though it increased slightly with larger sample sizes as more variants were included in the fitted model. With such a large number of variants, even a small false-positive rate can be problematic, and so combining selected variants with a ranking by *P* value from a GWAS is important. It is possible to do this with least angle regression (49) in lasso regression, but due to the large number of variants, a *P* value from GWAS is most convenient.

We also tested lasso regression on the same footing, which is the other extreme of the α setting in the elastic net. For smaller numbers of causal variants, performance was similar to that of the linear mixed model both in terms of power and false-positive rate, in some cases having slightly higher power. However, when the number of causal variants was higher, the amount of sparsity introduced was too high, reducing power below that of other methods (though false-positive rate was low in all settings for the same reason). As the number of causal variants is generally not known *a priori*, we would therefore always recommend the elastic net with a small α over the lasso.

These results show that the variants selected by the elastic net are causal at similar rates to those with GWAS methods. The elastic net’s selected variants can be used as an effective trade-off between the regimes of the two commonly used GWAS models, having higher power than the linear mixed model and a lower false-positive rate than fixed-effect models. As many bacterial GWAS results must be followed up with lab work, these results suggest a dual approach of variable selection with the elastic net followed by ranking results with the linear mixed model may be useful when the first set of variables selected is large. This is possible in a single step in pyseer.

### Whole-genome model of bacterial phenotypes enables heritability estimation and combination with association mapping.

**(i) Variant selection for pneumococcal antimicrobial resistances.** Next, we tested whether our method selects causal variants for some phenotypes where these are known and compared our results to those from GWAS approaches. First, we analyzed a well-studied phenotype and data set: sensitivity/resistance to β-lactams in the SPARC cohort of 603 S. pneumoniae genomes. Resistance is mainly conferred by allelic variation of three genes (*pbp1a*, *pbp2b*, and *pbp2x*), which are easily detected by most GWAS and machine learning methods with SNP calls as input (47, 50) Though the regions are always correctly identified, the specific variants detected are not identical between methods (51).

**FIG 3:**
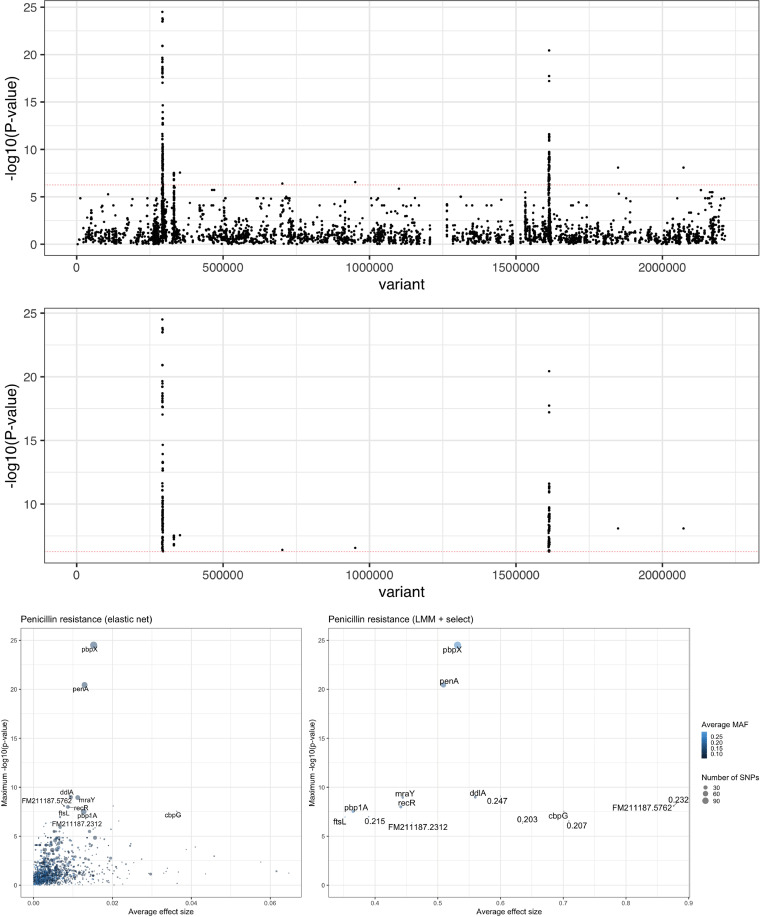
Elastic net and linear mixed model with SNP-based penicillin resistance. (Top) Manhattan plot of the selected elastic net variants, with a Bonferroni-adjusted significance threshold in red. The three biggest peaks are in the causal *pbp* genes. (Middle) The same result with the LMM, taking only those SNPs above the significance threshold. (Bottom) Summary of the genes selected by both methods (left, elastic net; right, LMM), maximum *P* value, and average absolute effect size within each gene.

Figure 3 shows the results of this analysis. With both the elastic net and linear mixed model plus cutoff, *penA*-*pbp2b* and *pbpX*-*pbp2x* are clearly the strongest hits. *pbp1a* is also selected by both methods, and while it can be seen on both Manhattan plots, it is slightly clearer in the gene summary plot for the elastic net, due to the larger number of SNPs selected in the gene. Taking hits with a *P* value above a threshold results in a very clean result for the linear mixed model (LMM) with this strongly selected phenotype; only a few noncausal genes are included, usually with only a few SNPs and much lower ranking than the causal genes. The elastic net selects many more noncausal SNPs across the MAF spectrum, though combining with *P* values and number of hits allows these to be effectively filtered. It should be noted that effect size does not appear to be an effective filtering criterion without taking into account minor allele frequency, which may have implications for other machine learning methods where a *P* value cannot easily be integrated. Both methods calculate comparable heritability estimates for this trait, for the LMM, *h*^2^ = 0.89, and for the elastic net, *h*^2^ = 0.81.

We then undertook a more challenging analysis, using unitigs to investigate antimicrobial resistances in the larger Maela cohort (3,162 S. pneumoniae genomes). We previously attempted GWAS on this data set using a fixed-effect GWAS model in the original description of our SEER software (12). We did not analyze tetracycline resistance or chloramphenicol resistance, as these are driven by single elements which were easily detected with the previous method. We instead used trimethoprim and erythromycin resistances. Trimethoprim resistance is expected to have two causal loci (*folA-dyr* and *folP*) due to being administered jointly with sulfamethoxazole as co-trimoxazole (52). Indeed, using our method on trimethoprim, the two causal loci are clearly identified and are the most highly ranked on a Manhattan plot (Fig. S3); applying sequence reweighting makes little difference to the result. Erythromycin resistance has multiple causal mechanisms (*ermB*, *mel*, and *mef*) which were not easily found in our previous attempt. Again, the erythromycin results contained many peaks in their Manhattan plots (see Fig. S4). When we mapped the unitigs directly to resistance genes, we found significant results in *ermB* (9 hits, minimum [min] *P* = 10^−47^), *mel* (12 hits, min *P* = 5 × 10^−42^), and *mef* (6 hits, min *P* = 5 × 10^−42^). While this was clearly more successful than our previous analysis, when considering the noise when mapping to a single reference, these causal mechanisms would not stand out (see Fig. S5). So, while our method reduced the computational burden of the analysis through the use of unitigs, it was not able to easily resolve the causal mechanisms in this challenging example. This suggests that both single variant tests and whole-genome models would struggle to arrive at true causal predictions under such circumstances. More flexible black box machine learning type approaches may help to improve prediction accuracy in these cases but were outside the scope of this study due to difficulty in interpreting the models in terms of causal variants.

10.1128/mBio.01344-20.3 FIG S3 Manhattan plots for trimethoprim resistance in S. pneumoniae showing locations of causal loci *folP* and *dyr*. Plotted variants are unitigs selected by the elastic net using sequence reweighting; *P* values are from the LMM. (a) Using sequence reweighting. (b) Without sequence reweighting. Download FIG S3, PDF file, 1.0 MB. Copyright © 2020 Lees et al. 2020 Lees et al. This content is distributed under the terms of the Creative Commons Attribution 4.0 International license.

10.1128/mBio.01344-20.4 FIG S4 Manhattan plots for erythromycin resistance in S. pneumoniae. Plotted variants are unitigs selected by the elastic net using sequence reweighting; *P* values are from the LMM. (a) Using sequence reweighting. (b) Without sequence reweighting. Download FIG S4, PDF file, 1.5 MB. Copyright © 2020 Lees et al. 2020 Lees et al. This content is distributed under the terms of the Creative Commons Attribution 4.0 International license.

10.1128/mBio.01344-20.5 FIG S5 Summary of genes with overlapping selected unitigs in the weighted GWAS. Each point is a gene, *x* axis is the average effect size (beta) of unitigs covering the locus, *y* axis is the minimum *P* value of any unitig in the locus; size relates to the total number of unitigs mapped to the gene, color is the average MAF of the mapped unitigs. (a) Using sequence reweighting. (b) Without sequence reweighting. Download FIG S5, PDF file, 0.7 MB. Copyright © 2020 Lees et al. 2020 Lees et al. This content is distributed under the terms of the Creative Commons Attribution 4.0 International license.

**(ii) Heritability and mapping of gonococcal resistance.** We also applied our method to a combined cohort of 1,595 Neisseria gonorrhoeae genomes where resistance to five different antibiotics has been measured. These data were previously used to do GWAS using an LMM, with selected loci then entering a reduced dimension epistasis analysis (53).

The mapping of resistance genes for these antibiotics using our approach was similar to this GWAS. The original analysis looked at ∼8,700 SNPs with a MAF of >0.5%; we used 5.3 × 10^5^ unitigs with a MAF of >1%. Azithromycin (AZI) had 4,612 unitigs selected, with the top hits mapping to the four 23S rRNA sequences in the genome. The original analysis only identified a SNP in one of these repeated rRNA sequences, likely due to the impossibility of mapping variation in these repeats at a single base level: this is an advantage of being able to report multiple mappings of sequences at the final stage. Cefixime (CFX) identified the *penA* region, as in the original analysis, and also suggested an association in the promoter of *opaD*. Ciprofloxacin (CIPRO) had hits throughout the genome, as in the original analysis and similar to the analysis of erythromycin described above—combining the LMM with the elastic net may reduce candidate regions in these cases. Penicillin (PEN) had a hit in the *porB* region, as in the original analysis, along with hits in *lgtE*, *mexB* (and efflux pump), and a prophage. Tetracycline (TET) similarly had a replicated hit in the *porB* region, along with the *cysN* promoter, an alternative *pilE* allele, and *rsmE* (a ribosomal methyltransferase). These Manhattan plots can be seen in Fig. S6. The WHO N strain (54) contains plasmids with *blaTEM* and *tetM*, causal for PEN and TET, respectively, to which we can also map unitigs rather than needing to recall variation with respect to this reference panel. This confirms further hits to these genes. Our method therefore broadly replicated the results from the LMM and added new candidate hits due to testing unitigs rather than just SNPs, as expected.

10.1128/mBio.01344-20.6 FIG S6 Manhattan plots for antimicrobial resistances in N. gonorrhoeae. Plotted variants are unitigs selected by the elastic net without sequence reweighting; *P* values are from the LMM. (a) Azithromycin. (b) Cefixime. (c) Ciprofloxacin. (d) Penicillin. (e) Tetracycline. Download FIG S6, PDF file, 2.6 MB. Copyright © 2020 Lees et al. 2020 Lees et al. This content is distributed under the terms of the Creative Commons Attribution 4.0 International license.

**FIG 4:**
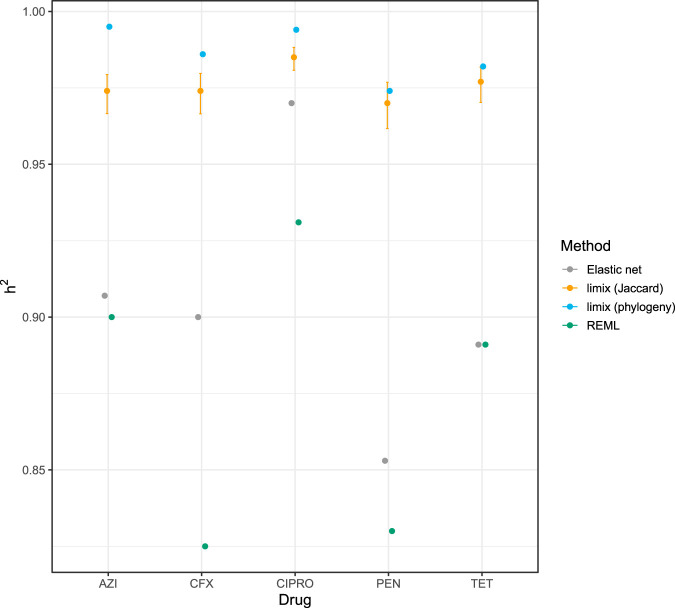
Heritability estimates for antibiotic resistance in the combined N. gonorrhoeae data set, using different methods. For each of the five antibiotic resistances measured in this data set, we report the narrow-sense heritability (*h*^2^) from our elastic net method and unitigs (gray), the limix method implemented in pyseer, using sequence distance (gold) or phylogeny (blue), and the restricted maximum likelihood (REML) approach used in the original publication (green). For limix estimates, 95% confidence intervals (CIs) were calculated with FIESTA (82). These are not shown for the phylogeny method as they span a range wider than the plot (0.11 to 1).

We also calculated the narrow sense heritability *h*^2^ using our elastic net and unitig method and compared these to those calculated with previous methods (Fig. 4). Our estimates were very similar to those of $h_{\text{SNP}}^{2}$ from the original paper, though consistently slightly higher (3% ± 7%), which may be a result of including more of the population variation through unitigs. Using a simple estimate of shared sequence content as the kinship matrix led to a likely overestimate of heritability. For these antibiotics, we expect high *h*^2^, approaching 1, as we discover further causal mechanisms and include them in the model (55). These estimates are consistent with this expectation but are difficult to evaluate quantitatively. It is challenging to evaluate the accuracy of heritability estimates, as the true biological value cannot easily be measured in bacteria and measurement via simulation is often circular, where methods used to generate the simulations necessarily perform best.

## DISCUSSION

In this paper, we developed a microbe-specific implementation of a machine learning tool and showed how this can be used to better understand the link between bacterial genetic variation and phenotypic variation. We argue that the sophistication of the prediction model itself is generally less important than three other factors: the data set itself, creating a method with careful genomic data management, and incorporating knowledge specific to bacterial populations. We addressed these issues as follows. Pangenomic variation was covered using a unitig definition of population variation, which we showed to be scalable, unlike k-mers, and better suited to analyzing accessory genome variation and inter-data set consistency, unlike SNPs. Population structure was accounted for explicitly using sequence reweighting and leave-one-strain-out cross-validation. We maintained a clear link between our resulting models and underlying genetics by combining linear models with a suite of tools to interpret the variants selected in the model. This had the further advantage that it significantly reduced the number of sequence elements to be processed after association. Using selected unitigs allowed for a much smoother use of the interactive plotting software phandango (56), which is one of the fastest ways to interpret bacterial GWAS results.

Our method is interpretable, and selected variants contain a high proportion of causal variants in simulations. We compared power and false-positive rate to selection with a *P* value threshold using simulations, and using real data from two species showed how this method can be combined with GWAS approaches to understand resistance and epistasis. LMMs are the best GWAS approach but mostly excel in cases where a trait is polygenic and has evolved multiple times, as is the case with many causal antibiotic resistance markers (11). If causal variants are more closely correlated with lineage, as may be the case for less strongly selected phenotypes, the LMM has reduced power, whereas the elastic net includes these groups of variants more effectively than previous fixed-effect models. This has recently been shown in an independent set of simulations (57). It is also worth noting that in highly clonal settings, the LMM can suffer from a very high false-positive rate unless the kinship matrix is carefully chosen (57, 58). Depending on the genetic architecture of the phenotype, which is rarely known *a priori*, one of these two methods may have a more desirable power/false-positive trade-off. The option to combine selection with the elastic net and ranking from the LMM appears to be useful in some challenging intermediate cases. An alternative approach is to associate LMM variants with lineages themselves marginally associated with the phenotype, as proposed in the bugwas package (11). The elastic net would be preferable where individual lineage associations are weak but causal, and the entire lineage block would be rejected.

We also obtain useful estimates of trait heritability, some of which show evidence that previous approaches may have overestimated this quantity. For the purposes of prediction, on a simple data set, we replicated the result that regularized linear models perform similarly to more complex deep learning methods. We also applied our method to a range of data sets from different species and phenotypes, including resistance, carriage duration, and virulence. Though our models generally performed well when measured on error rates, an experiment with models on three separate cohorts showed how accuracy falls outside the target data set. External data sets may have different strain compositions due to different biases toward more- or less-virulent strains, geographical separation, vaccine use, or antibiotic consumption in the population. We would reiterate the caveat that while these models can be useful, high accuracy on test data should not be taken as a general measure of confidence (9). Batch differences such as genotyping methods between cohorts exaggerate this problem, and so a consistent approach (such as the one we provide here) should be used. Unsurprisingly, curated resistance sets—the result of decades of research—still generally perform better, although even this *in silico* method loses accuracy between data sets (34). Less-well-understood and potentially polygenic phenotypes such as virulence offer an attractive target for our model, as we demonstrated on two streptococcal pathogens.

Along with these theoretical advances, our package has a number of practical advantages. All of the elements of our method are freely available, well documented, and part of a continuous unit-testing framework. Users can construct and evaluate models easily, without the need for programming experience, with options which retain the flexibility to modify the model parameters. There is no need for specialist hardware such as the graphics cards needed to fit large deep learning models. The models themselves are saved in a human-readable format, are easy to share and reuse, and have minimal hardware-specific requirements.

Our method does remain limited due to its reliance on the elastic net. Higher order interactions are difficult to include, as they make the size of the input space increase greatly, whereas in other machine learning approaches such as random forests, these are included naturally in the model structure. Simultaneous selection of both hyperparameters α and λ is more challenging due to a greatly increased search space in cross-validation, and so we rely on a heuristic selection of α. For larger data sets, the requirement to store the entire variant matrix in memory to fit the elastic net can easily exceed available memory. This could be solved by storing this matrix as a file on a disk and memory mapping this file, or reading rows only as needed (40). Our implementation cannot currently incorporate prior information on input variants, such as known association with antimicrobial resistances, though extension to either an ensemble model or Bayesian regression would be possible. As we have introduced a general framework for variant input/output in pyseer, we hope to include further machine learning approaches which allow trade-off of these advantages and disadvantages in the future.

The effect sizes from the elastic net do not necessarily indicate the significance of individual variants, as they are optimized for prediction; even after reweighting sequences, nonzero effects form a much larger set than true causal variants and are spread across the genome rather than specifically mapping to a causal region. The combination with *P* values from a well-performing GWAS method, such as the linear mixed model, helps if the user requires this interpretation. We would expect the same to be true for other machine learning methods and would generally caution against making a “GWAS-type” inference based on predictor importance or similar measures. We also did not perform a thorough comparison with other available machine learning methods; there are many choices, with well-known characteristics, which have previously been shown to perform similarly well at this task (10).

More broadly, we have considered techniques routinely used in the analysis of modern data sets, which are generated frequently with high-throughput methods. These can be adapted to perform fundamental tasks in bacterial genomics in ways which are useful and that scale with our ambitions to discover causal drivers and predict phenotypes from genome variation. Collections of high-quality whole-genome sequences are now available at a scale that would have been unfathomable just a few years ago. Many of these data sets are publicly available already, and many more are being generated from new larger projects and routine surveillance by public health agencies. Care must be taken to ensure the unique properties of bacterial populations are properly modeled and that we use appropriate measures of success. Complex models should be compared to simple models (59, 60), not just in terms of accuracy but also for their ability to look at underlying biology. In many cases, the limiting factor is unlikely to be model flexibility. With our pangenome-spanning penalized regression models, we hope to have made useful and usable contributions that respect these principles.

## MATERIALS AND METHODS

### Preparation of data sets.

Table 3 shows a summary of the data sets used in this paper. Sequence assemblies were available from the original publications, with the exception of one N. gonorrhoeae study (61). For this study, we downloaded the read data, removed adapter sequences with trimmomatic (62) v0.36, and assembled them with SPAdes v3.11 (63) using the --only-assembler and --careful options. For all data sets, we then called unitigs from each sample’s sequence assembly using a k-mer length of 31, and low-frequency unitigs (AF < 1%) were discarded. For the Massachusetts and tuberculosis (TB) data sets, additional genetic data were available. For the TB data set, we used the variant call matrix provided by the study’s authors (5). Not all phenotypes were available for all samples from this study. For the Massachusetts and Maela data sets, we used SNP calls from an earlier GWAS in this population (47). Where a split into training and test data was needed, this was performed at random in the ratio 2:1. When including a cluster assignment to account for population structure, we used the previous assignments from PopPUNK where available (64). For TB, we used the major lineage as the cluster. When MICs indicated antibiotic susceptibility, this phenotype was first log transformed before any downstream analysis. Other phenotypes were used as originally reported.

To generate simulated data used for testing power and false-discovery rate against a ground truth, we simulated phenotypes but used observed SNP genotype data from the Maela data set to ensure a realistic genetic model for bacterial population structure. Phenotypes were simulated using GCTA (16), either as continuous or as binary using a liability threshold model. Then, to assess the power of the methods with respect to the sample sizes, we randomly choose subsamples with 500, 1,000, 2,000, and 3,000 samples (with *h*^2^ fixed at 0.5). Separately, using 3,000 samples, *h*^2^ was varied at 0.1, 0.3, 0.6, and 0.9. We did not use fewer than 500 samples, as we expect a significant decrease in discovery power (12). LMM and fixed-effects models were run using default settings in pyseer (10 multidimensional scaling [MDS] components and kinship matrix estimated from shared distance to the root of a phylogeny). We used a fixed-effect size of 4, a relatively high effect size typical of that found for antimicrobial resistance SNPs, which have been found to range between roughly 2.5 to 7 for this data set (45), and higher than virulence effects which have been found to range between 0.1 and 1 in the Netherlands data set described above (43).

### Elastic net model.

We use a high-dimensional regression model which includes all genetic variants and covariates of interest $\overset{\to }{x}$. Typically, this is not possible using classical inference methods such as the least-squares estimator, as the number of genetic predictors *m* exceeds the number of samples *N*, leading to an underconstrained system. The elastic net defines such a function (33, 65), mixing L1 (lasso) and L2 (ridge) penalties, which is minimized with respect to the values of the intercept *b*_0_ and slopes $\overset{\to }{b}$:

$$\underset{b_{0},b}{\text{min}}\frac{1}{N}\overset{N}{\underset{i=1}{\sum }}w_{i}l{\left(y_{i},b_{0}+b^{T}x_{i}\right)}^{2}+\lambda \left[(1-\alpha )\|b{\|}_{2}^{2}/2+\alpha \|b{\|}_{1}\right],$$

where *N* is the number of samples, *w*_i_ are positive weights (with a sum equal to *N*), *l*() is the link function (linear or logit for continuous or binary phenotypes *y*_i_, respectively), λ > 0 is the magnitude of the penalty, and 0 < α < 1 is the amount of mixing between L1 and L2 penalties. Minimizing this function reduces the squared distance between predicted and observed values when λ = 0; as *λ* increases, predictors are shrunk toward zero to trade-off prediction accuracy with overfitting.

Given the strong linkage disequilibrium present in bacterial populations (11, 66), many genetic variants are strongly correlated across long distances. Randomly selecting a single representative variant from such a group will likely lead to greatly varied biological conclusions, and so including the entire block with suitable lineage annotation is preferred (11). For this reason, the elastic net has been shown to be especially useful when the variables are dependent (33). We compare this selection with the lasso in our simulations. The value of α can be changed by the user, should they wish to opt for a sparser model.

Two important parameters which are not directly set using the data are λ and α. The amount of penalty λ is set by cross-validation, using a default of 10-fold to pick the value of λ, which maximizes the cross-validated *R*^2^ value of the model over the test data. The user can change the number of folds. The amount of mixing (α) between L1 penalties, which lead to sparse predictive models with mostly zero valued slopes, and L2 penalties, which lead to models with shrunken but nonzero predictors, could also be chosen by cross-validation to maximize prediction accuracy. However, we propose users choose a value of around 0.01 throughout (all experiments reported here use α = 0.01). A sparse model is preferred for prediction for speed and easier consistency between populations, but this leads to a loss of power in the context of GWAS, as potentially causally related predictors may be removed from the model (Fig. 2 and S2 in the supplemental material). Introducing even a small L1 penalty in this way removes a large fraction of variants unconnected with the phenotype.

By virtue of the fact that all pangenomic variation enters this model, if a linear additive model of heritability is assumed, which it typically is in bacterial GWAS (44, 45, 67), the value of *R*^2^ calculated from the fit of the elastic net,

R2=1−‖y−Xβ^enet‖2‖y−y¯‖2,

also serves as an estimate of the narrow-sense heritability *h*^2^. As *R*^2^ measures the variance explained by the model’s predictors, in this case all genetic features, this is equivalent to the proportion of phenotypic variance $\sigma_{p}{}^{2}$ explained by genetic variation $\sigma_{g}{}^{2}$, the definition of *h*^2^. This provides a lower bound on *h*^2^, because the Lasso-type estimator is biased (68), and it tends to shrink some coefficients with weak effects toward zero, though these weak effects may have a significant effect on the trait variability.

### Efficiently modeling the entire pangenome.

Bacterial populations vary greatly in their sequence content, and mapping short variation within their core genes (coding sequences shared by all members of the population) is generally insufficient to capture all of the variation within the samples. In particular, accessory gene content has been shown to be associated with core variation (64), be associated with clinically relevant phenotypes (12, 69), and be useful for predicting the evolution of the population (70, 71).

Early bacterial GWAS methods used k-mers, sequence words of fixed or variable length, to assay variation throughout the population independent of gene annotation or variant calling method (11, 12, 35). The set of common k-mers (1% to 99% frequency) is vast, particularly in the large and genetically diverse populations which are most amenable to GWAS. Efforts to model all of these sequence elements simultaneously are potentially computationally intractable, as these words will not fit in main memory and model fitting takes an extremely long time. In the S. pneumoniae data sets, we observed a 13- to 18-fold reduction in the number of unitigs compared to that of k-mers.

We used two techniques to circumvent this issue while still including as much pangenomic variation as possible. Following the idea of screening methods in ultrahigh-dimensional data (72, 73), we used the absolute value of the sample correlation as a screening criterion for each variant:

$$\left|cor(y,x)|=|〈\overset{¯}{y},\overset{¯}{x}〉|=|\Sigma \overset{¯}{y_{i}}\;\overset{¯}{x_{i}}\right|$$

where $\overset{¯}{y}$ and $\overset{¯}{x}$ are the standardizations of *y* and *x* (defined as phenotype and variant as above) such that

$$\sum {\bar{x}}_{i}=0,\sum {\bar{x}}_{i}^{2}=1,\sum {\bar{y}}_{i}=0,\sum {\bar{y}}_{i}^{2}=1.$$

This is the “correlation filter.” Using a single threshold on the mean value for this correlation would lead to a large number of variants being removed before modeling, which is appealing computationally but, in our simulations, led to a loss of power. We instead removed the lowest quartile, which maintained power but did not reduce model size as much. The size of the quantile to remove can be set by the user.

We also followed the method used in DBGWAS (36), which after counting fixed-length k-mers, constructs a compressed de Bruijn graph (DBG) of the population. Nodes in this compressed graph are extensions of adjacent k-mers in the raw graph with the same population frequency vector and whose sequences are referred to as unitigs. These unitigs greatly reduce the redundancy present in raw k-mer counts by combining those with the same patterns and are generally easier to functionally interpret due to their longer length. We followed the same method as step 1 of the DBGWAS package, which uses the GATB library to construct a compressed DBG (74), and then reported frequency vectors of each unitig/node and unique pattern in a format readable by pyseer. We used a k-mer length of 31 throughout to count unitigs, as this was previously shown to maximize association power (36). This length can also be set by the user. We reimplemented this approach as a standalone package (https://github.com/johnlees/unitig-counter), also including tools to extend unitigs by traversing neighboring nodes in the graph, and calculate distances between unitigs based on the graph using Dijkstra’s algorithm.

### Incorporating population structure.

Population structure causes correlations between the genetic variants (unitigs) that make up $\overset{\to }{x}$. As all of these enter our model together, this effect may be implicitly controlled for without the need for further correction. We also wished to compare this to the use of an explicit correction term to test which approach is more effective. This can be included in the modeling step by a combination of three approaches: use of extra predictors in $\overset{\to }{x}$ which account for population structure, modifying the per-sample weights *w*_i_, or by changing the folds used in cross-validation. We discuss each of these in turn.

Fixed-effect models typically use a principal components-type analysis to include the main axes of variation in the population as covariates. In a new data set, projection of variation onto these existing axes could be used but would require large overlap between variant calls in each data set to be accurate. Random-effect models use a kinship matrix to include the sample covariance matrix in each association. For a new data set, this would require calculation of covariance against the original data set, which reduces model portability.

We therefore opted to use a definition of population structure which does not introduce extra predictors. This makes application of the model more straightforward in new data sets. The use of a definition of clusters which naturally extends to new populations, which may have very different strain frequencies and/or large numbers of novel clusters, further increases robustness in the face of between-data set variation. Any method which produces discrete cluster membership definitions independent of cluster frequency is suitable for this purpose, such as sequence type, clonal complex, or percentage identity cutoff. We opted to use the “strain” definition provided by the PopPUNK software throughout our analysis due to its speed and biological basis (64) However, our implementation allows any preferred definition of cluster membership to be used.

Cluster membership for each sample x is defined as C_i_(x), which is 1 if x is in cluster i or 0 otherwise. The model is fit to all the data except the first cluster, and then its accuracy is tested on the cluster not used in the fitting. This is repeated for every cluster to find the value of λ which maximizes fit accuracy over all these fits. This may be referred to as leave-one-strain-out (LOSO) cross-validation or leave-one-cluster-out (LOCO) cross-validation. This is more realistic than random selection of folds, as random samples would maintain relative strain frequencies between training and test data, whereas new populations usually vary greatly in their genetic background (1, 10).

Furthermore, we added the sequence-reweighting option in pyseer, which defines the sample weights as being inversely proportional to the cluster/strain size:

$$\begin{array}{l} \frac{1}{u_{i}}=\overset{N}{\underset{j=1}{\sum }}\left[j\in C_{i}\right]\\ w_{i}=u_{i}\times \frac{N}{\sum_{j=1}^{N}u_{j}}\text{.} \end{array}$$

This sequence reweighting is a commonly used definition in epistasis methods such as direct coupling analysis and correlation-based approaches, which have recently been successfully applied to genome-wide variation in bacterial populations (38, 39, 75).

### Phenotypic prediction while maximizing consistency between data sets.

Prediction of unobserved phenotypes *y*_i_ is achieved by forming a linear predictor of nonzero slopes in ${\vec{b}}_{i}$:

$$b_{0}+b^{T}x_{i},$$

to which the appropriate link function *l*() is then applied to convert into a probability (which can be converted into a binary outcome using a threshold cutoff).

Predictors which are missing can either be ignored (set *x_i_* = 0) or imputed (set $x_{i}=\bar{x}$, where $\bar{x}$ is the allele frequency in the original data set). For the unitig caller approach described below, a missing call means a genuine absence in the data, and so we used the former approach. For variant calling methods where missing calls may be artifactual (such as SNP calls), we use the mean value imputation approach. The option –ignore-missing can be used to control this behavior manually.

To apply fitted models to new populations, consistency in the construction of the matrix of genomic variants $\vec{x}$ between data sets is important to maximize prediction accuracy. This can be highly challenging for core SNPs due to the many possible methods and individual options to extract these from sequence reads. Sequence element presence or absence is simple to define, and so it is easier to enforce consistency of input between data sets. The main issue with using unitigs here is that DBGs of different sample sets will have different sequences at their nodes and, therefore, nonoverlapping unitig calls. To solve this issue, we defined unitig sequences once in the training population. Rather than building a new DBG in test data sets, we instead checked for the presence of unitigs previously defined in the training population. To do this efficiently, we created and saved an Ferragina and Manzini (FM) index for each input sequence (76), which is a substring index capable of supporting fast text searches, which we performed in parallel for each unitig query. We implemented this as a separate package unitig-caller (https://github.com/johnlees/unitig-caller) in C++ using the SeqAn library (77). An alternative implementation of both “counting” and “calling” unitigs is possible by constructing and querying sequences using a single population DBG. We implemented this in the same package by creating an interface to the Bifrost method (78).

For continuous phenotypes, we report *R*^2^ to describe prediction accuracy. This is more difficult to interpret with binary phenotypes, especially in the presence of class imbalance and when the imbalance deviates from the population-wide prevalence. For binary phenotypes, we found that reporting positive statistics such as sensitivity and specificity, or especially area under the curve (AUC), led to reports in the top decile for almost all data sets and methods and were harder to intuitively compare between different models and data sets. We therefore report the false-negative rate and false-positive rate along with the totals selected.

### Implementation.

We implemented the association model in version 1.3.2 of the pyseer bacterial GWAS package, which is written in python (79). This takes care of reading variants in many common formats, including the output from unitig-counter, as well as providing tools to help interpret associated sequences. We used python bindings to the fortran glmnet package to actually fit the model, as the use of warm-starts more efficiently solves the above equation at an array of values of λ (65). Cross-validation, parallelized if requested by the user, is used to select the value of λ with the greatest *R*^2^ value, as defined above.

Variant matrices are potentially very large, and so to optimize speed and memory use, we read these into a sparse matrix structure. Variants with allele frequency of >50% have their genotypes flipped to increase sparsity—these sites are flipped back during prediction. This sparse structure can be saved to disk to avoid repeated parsing of variant input files. Only haploid variant calls (0/1), allele frequency, and sample order are saved in this file. After extracting the nonzero coefficients from the fitted elastic net, the input variant file is reread with minimal parsing to output information about the selected variants. A SHA256 hash of the input file is calculated to ensure consistency with the original input file.

The fitted models are saved as an associative array, with variant names as keys (either sequence or alleles combined with chromosome and position) and allele frequencies and fitted slopes as values. New variant call files are read with minimal parsing to extract just those sites which appear in the model, and at the end-of-file, the appropriate imputation procedure is applied to model terms which were not found. This allows both rapid prediction in large new data sets and an easy and portable way to share predictive models.

For fitted models, slopes, *P* values (adjusted by any of pyseer’s other models), and allele frequencies are included in the output. Where the true phenotype is known, prediction accuracy is reported using *R*^2^ and a confusion matrix if the phenotype is binary. If clusters were provided, accuracy statistics for within each cluster are also included in the output.

We used the tools within pyseer to interpret unitigs. Specifically, we used bwa-mem with the shortest possible seed to map unitig sequences to a single reference genome (Streptococcus pneumoniae ATCC 700669 [80]; Neisseria gonorrhoeae FA1090 [81], and WHO_N [54]). Bonferroni corrections were calculated by using the number of unique unitig patterns as the number of tests.

The new code in pyseer includes automated tests and unit tests we wrote using test data distributed with the package. Documentation and a tutorial are available online (https://pyseer.readthedocs.io/en/master/predict.html).

### Availability of data and materials.

The pyseer package is available as source code at the GitHub repository (https://github.com/mgalardini/pyseer; Apache 2.0 license), documented on readthedocs (http://pyseer.readthedocs.io/), and available for install on bioconda (https://anaconda.org/bioconda/pyseer). The unitig-counter package is available at the GitHub repository (https://github.com/johnlees/unitig-counter; AGPL 3.0 license) and available for install on bioconda (https://anaconda.org/bioconda/unitig-counter). The unitig-caller package is available at the GitHub repository (https://github.com/johnlees/unitig-caller; Apache 2.0 license) and available for install on bioconda (https://anaconda.org/bioconda/unitig-caller).

## References

[1] Gladstone RA, Lo SW, Lees JA, Croucher NJ, van Tonder AJ, Corander J, Page AJ, Marttinen P, Bentley LJ, Ochoa TJ, Ho PL, Du Plessis M, Cornick JE, Kwambana-Adams B, Benisty R, Nzenze SA, Madhi SA, Hawkins PA, Everett DB, Antonio M, Dagan R, Klugman KP, von Gottberg A, McGee L, Breiman RF, Bentley SD, Global Pneumococcal Sequencing Consortium. 2019 International genomic definition of pneumococcal lineages, to contextualise disease, antibiotic resistance and vaccine impact. EBioMedicine 43:338–346. doi:10.1016/j.ebiom.2019.04.021. 31003929 PMC6557916

[2] CRyPTIC Consortium and the 100,000 Genomes Project, Allix-Béguec C, Arandjelovic I, Bi L, Beckert P, Bonnet M, Bradley P, Cabibbe AM, Cancino-Muñoz I, Caulfield MJ, Chaiprasert A, Cirillo DM, Clifton DA, Comas I, Crook DW, De Filippo MR, de Neeling H, Diel R, Drobniewski FA, Faksri K, Farhat MR, Fleming J, Fowler P, Fowler TA, Gao Q, Gardy J, Gascoyne-Binzi D, Gibertoni-Cruz A-L, Gil-Brusola A, Golubchik T, Gonzalo X, Grandjean L, He G, Guthrie JL, Hoosdally S, Hunt M, Iqbal Z, Ismail N, Johnston J, Khanzada FM, Khor CC, Kohl TA, Kong C, Lipworth S, Liu Q, Maphalala G, Martinez E, Mathys V, Merker M, Miotto P, 2018 Prediction of susceptibility to first-line tuberculosis drugs by DNA sequencing. N Engl J Med 379:1403–1415. doi:10.1056/NEJMoa1800474. 30280646 PMC6121966

[3] Lupolova N, Dallman TJ, Holden NJ, Gally DL 2017 Patchy promiscuity: machine learning applied to predict the host specificity of *Salmonella enterica* and *Escherichia coli*. Microb Genom 3:e000135. doi:10.1099/mgen.0.000135. 29177093 PMC5695212

[4] Moradigaravand D, Palm M, Farewell A, Mustonen V, Warringer J, Parts L 2018 Prediction of antibiotic resistance in Escherichia coli from large-scale pan-genome data. PLoS Comput Biol 14:e1006258. doi:10.1371/journal.pcbi.1006258. 30550564 PMC6310291

[5] Chen ML, Doddi A, Royer J, Freschi L, Schito M, Ezewudo M, Kohane IS, Beam A, Farhat M 2019 Beyond multidrug resistance: leveraging rare variants with machine and statistical learning models in *Mycobacterium tuberculosis* resistance prediction. EBioMedicine 43:356–369. doi:10.1016/j.ebiom.2019.04.016. 31047860 PMC6557804

[6] Khaledi A, Weimann A, Schniederjans M, Asgari E, Kuo T-H, Oliver A, Cabot G, Kola A, Gastmeier P, Hogardt M, Jonas D, Mofrad MRK, Bremges A, McHardy AC, Häussler S 2020 Fighting antimicrobial resistance in Pseudomonas aeruginosa with machine learning-enabled molecular diagnostics. EMBO Mol Med 12:e10264. doi:10.15252/emmm.201910264. 32048461 PMC7059009

[7] Naidenov BV, Lim A, Willyerd K, Torres NJ, Johnson WL, Hwang HJ, Hoyt PR, Gustafson JE, Chen C 2019 Pan-genomic and polymorphic driven prediction of antibiotic resistance in *Elizabethkingia*. Front Microbiol 10:1446. doi:10.3389/fmicb.2019.01446. 31333599 PMC6622151

[8] Pataki BÁ, Matamoros S, van der Putten BCL, Remondini D, Giampieri E, Aytan-Aktug D, Hendriksen RS, Lund O, Csabai I, Schultsz C 17 1 2020 Understanding and predicting ciprofloxacin minimum inhibitory concentration in *Escherichia coli* with machine learning. bioRxiv doi:10.1101/806760. PMC7490380 32929164

[9] Wheeler NE 2019 Tracing outbreaks with machine learning. Nat Rev Microbiol 17:269. doi:10.1038/s41579-019-0153-1. 30742026

[10] Hicks AL, Wheeler N, Sánchez-Busó L, Rakeman JL, Harris SR, Grad YH 2019 Evaluation of parameters affecting performance and reliability of machine learning-based antibiotic susceptibility testing from whole genome sequencing data. PLoS Comput Biol 15:e1007349. doi:10.1371/journal.pcbi.1007349. 31479500 PMC6743791

[11] Earle SG, Wu C-H, Charlesworth J, Stoesser N, Gordon NC, Walker TM, Spencer CCA, Iqbal Z, Clifton DA, Hopkins KL, Woodford N, Smith EG, Ismail N, Llewelyn MJ, Peto TE, Crook DW, McVean G, Walker AS, Wilson DJ 2016 Identifying lineage effects when controlling for population structure improves power in bacterial association studies. Nat Microbiol 1:16041. doi:10.1038/nmicrobiol.2016.41. 27572646 PMC5049680

[12] Lees JA, Vehkala M, Välimäki N, Harris SR, Chewapreecha C, Croucher NJ, Marttinen P, Davies MR, Steer AC, Tong SYC, Honkela A, Parkhill J, Bentley SD, Corander J 2016 Sequence element enrichment analysis to determine the genetic basis of bacterial phenotypes. Nat Commun 7:12797. doi:10.1038/ncomms12797. 27633831 PMC5028413

[13] Collins C, Didelot X 2018 A phylogenetic method to perform genome-wide association studies in microbes that accounts for population structure and recombination. PLoS Comput Biol 14:e1005958. doi:10.1371/journal.pcbi.1005958. 29401456 PMC5814097

[14] Spain SL, Barrett JC 2015 Strategies for fine-mapping complex traits. Hum Mol Genet 24:R111–R119. doi:10.1093/hmg/ddv260. 26157023 PMC4572002

[15] Huang H, Fang M, Jostins L, Umićević Mirkov M, Boucher G, Anderson CA, Andersen V, Cleynen I, Cortes A, Crins F, D'Amato M, Deffontaine V, Dmitrieva J, Docampo E, Elansary M, Farh KK-H, Franke A, Gori A-S, Goyette P, Halfvarson J, Haritunians T, Knight J, Lawrance IC, Lees CW, Louis E, Mariman R, Meuwissen T, Mni M, Momozawa Y, Parkes M, Spain SL, Théâtre E, Trynka G, Satsangi J, van Sommeren S, Vermeire S, Xavier RJ, International Inflammatory Bowel Disease Genetics Consortium, Weersma RK, Duerr RH, Mathew CG, Rioux JD, McGovern DPB, Cho JH, Georges M, Daly MJ, Barrett JC 2017 Fine-mapping inflammatory bowel disease loci to single-variant resolution. Nature 547:173–178. doi:10.1038/nature22969. 28658209 PMC5511510

[16] Yang J, Lee SH, Goddard ME, Visscher PM 2011 GCTA: a tool for genome-wide complex trait analysis. Am J Hum Genet 88:76–82. doi:10.1016/j.ajhg.2010.11.011. 21167468 PMC3014363

[17] Vilhjálmsson BJ, Yang J, Finucane HK, Gusev A, Lindström S, Ripke S, Genovese G, Loh P-R, Bhatia G, Do R, Hayeck T, Won H-H, Schizophrenia Working Group of the Psychiatric Genomics Consortium, Discovery, Biology, and Risk of Inherited Variants in Breast Cancer (DRIVE) study, Kathiresan S, Pato M, Pato C, Tamimi R, Stahl E, Zaitlen N, Pasaniuc B, Belbin G, Kenny EE, Schierup MH, De Jager P, Patsopoulos NA, McCarroll S, Daly M, Purcell S, Chasman D, Neale B, Goddard M, Visscher PM, Kraft P, Patterson N, Price AL 2015 Modeling linkage disequilibrium increases accuracy of polygenic risk scores. Am J Hum Genet 97:576–592. doi:10.1016/j.ajhg.2015.09.001. 26430803 PMC4596916

[18] Halevy A, Norvig P, Pereira F 2009 The unreasonable effectiveness of data. IEEE Intell Syst 24:8–12. doi:10.1109/MIS.2009.36.

[19] Chen KM, Cofer EM, Zhou J, Troyanskaya OG 2019 Selene: a PyTorch-based deep learning library for sequence data. Nat Methods 16:315–318. doi:10.1038/s41592-019-0360-8. 30923381 PMC7148117

[20] Zou J, Huss M, Abid A, Mohammadi P, Torkamani A, Telenti A 2019 A primer on deep learning in genomics. Nat Genet 51:12–18. doi:10.1038/s41588-018-0295-5. 30478442 PMC11180539

[21] Drouin A, Letarte G, Raymond F, Marchand M, Corbeil J, Laviolette F 2019 Interpretable genotype-to-phenotype classifiers with performance guarantees. Sci Rep 9:4071. doi:10.1038/s41598-019-40561-2. 30858411 PMC6411721

[22] Thompson A 1997 An evolved circuit, intrinsic in silicon, entwined with physics, p 390–405. Evolvable systems: from biology to hardware. Springer, Berlin, Germany.

[23] Ribeiro MT, Singh S, Guestrin C 16 2 2016 “Why should i trust you?”: Explaining the predictions of any classifier. arXiv [csLG] https://arxiv.org/abs/1602.04938.

[24] Duncan L, Shen H, Gelaye B, Meijsen J, Ressler K, Feldman M, Peterson R, Domingue B 2019 Analysis of polygenic risk score usage and performance in diverse human populations. Nat Commun 10:3328. doi:10.1038/s41467-019-11112-0. 31346163 PMC6658471

[25] Waldron L, Pintilie M, Tsao M-S, Shepherd FA, Huttenhower C, Jurisica I 2011 Optimized application of penalized regression methods to diverse genomic data. Bioinformatics 27:3399–3406. doi:10.1093/bioinformatics/btr591. 22156367 PMC3232376

[26] Waldmann P, Mészáros G, Gredler B, Fuerst C, Sölkner J 2013 Evaluation of the lasso and the elastic net in genome-wide association studies. Front Genet 4:270. doi:10.3389/fgene.2013.00270. 24363662 PMC3850240

[27] Yi H, Breheny P, Imam N, Liu Y, Hoeschele I 2015 Penalized multimarker vs. single-marker regression methods for genome-wide association studies of quantitative traits. Genetics 199:205–222. doi:10.1534/genetics.114.167817. 25354699 PMC4286685

[28] Abraham G, Kowalczyk A, Zobel J, Inouye M 2013 Performance and robustness of penalized and unpenalized methods for genetic prediction of complex human disease. Genet Epidemiol 37:184–195. doi:10.1002/gepi.21698. 23203348

[29] Gianola D, Fariello MI, Naya H, Schön C-C 2016 Genome-wide association studies with a genomic relationship matrix: a case study with wheat and Arabidopsis. G3 (Bethesda) 6:3241–3256. doi:10.1534/g3.116.034256. 27520956 PMC5068945

[30] Wu TT, Chen YF, Hastie T, Sobel E, Lange K 2009 Genome-wide association analysis by lasso penalized logistic regression. Bioinformatics 25:714–721. doi:10.1093/bioinformatics/btp041. 19176549 PMC2732298

[31] Buzdugan L, Kalisch M, Navarro A, Schunk D, Fehr E, Bühlmann P 2016 Assessing statistical significance in multivariable genome wide association analysis. Bioinformatics 32:1990–2000. doi:10.1093/bioinformatics/btw128. 27153677 PMC4920127

[32] Brzyski D, Peterson CB, Sobczyk P, Candès EJ, Bogdan M, Sabatti C 2017 Controlling the rate of GWAS false discoveries. Genetics 205:61–75. doi:10.1534/genetics.116.193987. 27784720 PMC5223524

[33] Zou H, Hastie T 2005 Regularization and variable selection via the elastic net. J R Stat Soc Series B Stat Methodol 67:301–320. doi:10.1111/j.1467-9868.2005.00503.x.

[34] Doyle RM, O’Sullivan DM, Aller SD, Bruchmann S, Clark T, Pelegrin AC, Cormican M, Benavente ED, Ellington MJ, McGrath E, Motro Y, Nguyen TPT, Phelan J, Shaw LP, Stabler RA, van Belkum A, van Dorp L, Woodford N, Moran-Gilad J, Huggett JF, Harris KA 2020 Discordant bioinformatic predictions of antimicrobial resistance from whole-genome sequencing data of bacterial isolates: an inter-laboratory study. Microb Genom 6:e000335. doi:10.1099/mgen.0.000335. PMC7067211 32048983

[35] Sheppard SK, Didelot X, Meric G, Torralbo A, Jolley KA, Kelly DJ, Bentley SD, Maiden MCJ, Parkhill J, Falush D 2013 Genome-wide association study identifies vitamin B5 biosynthesis as a host specificity factor in *Campylobacter*. Proc Natl Acad Sci U S A 110:11923–11927. doi:10.1073/pnas.1305559110. 23818615 PMC3718156

[36] Jaillard M, Lima L, Tournoud M, Mahé P, van Belkum A, Lacroix V, Jacob L 2018 A fast and agnostic method for bacterial genome-wide association studies: bridging the gap between k-mers and genetic events. PLoS Genet 14:e1007758. doi:10.1371/journal.pgen.1007758. 30419019 PMC6258240

[37] Kavvas ES, Catoiu E, Mih N, Yurkovich JT, Seif Y, Dillon N, Heckmann D, Anand A, Yang L, Nizet V, Monk JM, Palsson BO 2018 Machine learning and structural analysis of *Mycobacterium tuberculosis* pan-genome identifies genetic signatures of antibiotic resistance. Nat Commun 9:4306. doi:10.1038/s41467-018-06634-y. 30333483 PMC6193043

[38] Puranen S, Pesonen M, Pensar J, Xu YY, Lees JA, Bentley SD, Croucher NJ, Corander J 2018 SuperDCA for genome-wide epistasis analysis. Microb Genom 4:e000184. doi:10.1099/mgen.0.000184. PMC6096938 29813016

[39] Pensar J, Puranen S, Arnold B, MacAlasdair N, Kuronen J, Tonkin-Hill G, Pesonen M, Xu Y, Sipola A, Sánchez-Busó L, Lees JA, Chewapreecha C, Bentley SD, Harris SR, Parkhill J, Croucher NJ, Corander J 2019 Genome-wide epistasis and co-selection study using mutual information. Nucleic Acids Res 47:e112. doi:10.1093/nar/gkz656. 31361894 PMC6765119

[40] Qian J, Du W, Tanigawa Y, Aguirre M, Tibshirani R, Rivas MA, Hastie T 7 5 2019 A fast and flexible algorithm for solving the lasso in large-scale and ultrahigh-dimensional problems. bioRxiv doi:10.1101/630079.

[41] Mahé P, Tournoud M 2018 Predicting bacterial resistance from whole-genome sequences using k-mers and stability selection. BMC Bioinformatics 19:383. doi:10.1186/s12859-018-2403-z. 30332990 PMC6192184

[42] Lehtinen S, Blanquart F, Croucher NJ, Turner P, Lipsitch M, Fraser C 2017 Evolution of antibiotic resistance is linked to any genetic mechanism affecting bacterial duration of carriage. Proc Natl Acad Sci U S A 114:1075–1080. doi:10.1073/pnas.1617849114. 28096340 PMC5293062

[43] Lo SW, Gladstone RA, van Tonder AJ, Lees JA, Du Plessis M, Benisty R, Givon-Lavi N, Hawkins PA, Cornick JE, Kwambana-Adams B, Law PY, Ho PL, Antonio M, Everett DB, Dagan R, von Gottberg A, Klugman KP, McGee L, Breiman RF, Bentley SD, Global Pneumococcal Sequencing Consortium. 2019 Pneumococcal lineages associated with serotype replacement and antibiotic resistance in childhood invasive pneumococcal disease in the post-PCV13 era: an international whole-genome sequencing study. Lancet Infect Dis 19:759–769. doi:10.1016/S1473-3099(19)30297-X. 31196809 PMC7641901

[44] Lees JA, Croucher NJ, Goldblatt D, Nosten F, Parkhill J, Turner C, Turner P, Bentley SD 2017 Genome-wide identification of lineage and locus specific variation associated with pneumococcal carriage duration. Elife 6:e26255. doi:10.7554/eLife.26255. 28742023 PMC5576492

[45] Lees JA, Ferwerda B, Kremer PHC, Wheeler NE, Serón MV, Croucher NJ, Gladstone RA, Bootsma HJ, Rots NY, Wijmega-Monsuur AJ, Sanders EAM, Trzciński K, Wyllie AL, Zwinderman AH, van den Berg LH, van Rheenen W, Veldink JH, Harboe ZB, Lundbo LF, de Groot L, van Schoor NM, van der Velde N, Ängquist LH, Sørensen TIA, Nohr EA, Mentzer AJ, Mills TC, Knight JC, Du Plessis M, Nzenze S, Weiser JN, Parkhill J, Madhi S, Benfield T, von Gottberg A, van der Ende A, Brouwer MC, Barrett JC, Bentley SD, van de Beek D 2019 Joint sequencing of human and pathogen genomes reveals the genetics of pneumococcal meningitis. Nat Commun 10:2176. doi:10.1038/s41467-019-09976-3. 31092817 PMC6520353

[46] Davies MR, McIntyre L, Mutreja A, Lacey JA, Lees JA, Towers RJ, Duchêne S, Smeesters PR, Frost HR, Price DJ, Holden MTG, David S, Giffard PM, Worthing KA, Seale AC, Berkley JA, Harris SR, Rivera-Hernandez T, Berking O, Cork AJ, Torres R, Lithgow T, Strugnell RA, Bergmann R, Nitsche-Schmitz P, Chhatwal GS, Bentley SD, Fraser JD, Moreland NJ, Carapetis JR, Steer AC, Parkhill J, Saul A, Williamson DA, Currie BJ, Tong SYC, Dougan G, Walker MJ 2019 Atlas of group A streptococcal vaccine candidates compiled using large-scale comparative genomics. Nat Genet 51:1295. doi:10.1038/s41588-019-0482-z. 31324894

[47] Chewapreecha C, Marttinen P, Croucher NJ, Salter SJ, Harris SR, Mather AE, Hanage WP, Goldblatt D, Nosten FH, Turner C, Turner P, Bentley SD, Parkhill J 2014 Comprehensive identification of single nucleotide polymorphisms associated with beta-lactam resistance within pneumococcal mosaic genes. PLoS Genet 10:e1004547. doi:10.1371/journal.pgen.1004547. 25101644 PMC4125147

[48] Nebenzahl-Guimaraes H, van Laarhoven A, Farhat MR, Koeken VA, Mandemakers JJ, Zomer A, van Hijum SA, Netea MG, Murray M, van Crevel R, van Soolingen D 2017 Transmissible *Mycobacterium tuberculosis* strains share genetic markers and immune phenotypes. Am J Respir Crit Care Med 195:1519–1527. doi:10.1164/rccm.201605-1042OC. 27997216 PMC5803666

[49] Lockhart R, Taylor J, Tibshirani RJ, Tibshirani R 2014 A significance test for the lasso. Ann Stat 42:413–468. doi:10.1214/13-AOS1175. 25574062 PMC4285373

[50] Li Y, Metcalf BJ, Chochua S, Li Z, Gertz RE Jr, Walker H, Hawkins PA, Tran T, Whitney CG, McGee L, Beall BW 2016 Penicillin-binding protein transpeptidase signatures for tracking and predicting β-lactam resistance levels in *Streptococcus pneumoniae*. mBio 7:e00756-16. doi:10.1128/mBio.00756-16. 27302760 PMC4916381

[51] Dewé TCM, D’Aeth JC, Croucher NJ 2019 Genomic epidemiology of penicillin-non-susceptible *Streptococcus pneumoniae*. Microb Genom 5:e000305. doi:10.1099/mgen.0.000305. PMC6861860 31609685

[52] Rossolini GM, Arena F, Giani T 2017 138. Mechanisms of antibacterial resistance, p 1181–1196. *In* Cohen J, Powderly WG, Opal SM (ed), Infectious diseases (4th Edition). Elsevier, Amsterdam, Netherlands.

[53] Schubert B, Maddamsetti R, Nyman J, Farhat MR, Marks DS 2019 Genome-wide discovery of epistatic loci affecting antibiotic resistance in *Neisseria gonorrhoeae* using evolutionary couplings. Nat Microbiol 4:328–338. doi:10.1038/s41564-018-0309-1. 30510172 PMC6663919

[54] Unemo M, Golparian D, Sánchez-Busó L, Grad Y, Jacobsson S, Ohnishi M, Lahra MM, Limnios A, Sikora AE, Wi T, Harris SR 2016 The novel 2016 WHO *Neisseria gonorrhoeae* reference strains for global quality assurance of laboratory investigations: phenotypic, genetic and reference genome characterization. J Antimicrob Chemother 71:3096–3108. doi:10.1093/jac/dkw288. 27432602 PMC5079299

[55] Ma KC, Mortimer TD, Hicks AL, Wheeler NE, Sánchez-Busó L, Golparian D, Taiaroa G, Rubin DHF, Wang Y, Williamson DA, Unemo M, Harris SR, Grad YH 8 1 2020 Increased antibiotic susceptibility in *Neisseria gonorrhoeae* through adaptation to the cervical environment. bioRxiv doi:10.1101/2020.01.07.896696. PMC7431566 32807804

[56] Hadfield J, Croucher NJ, Goater RJ, Abudahab K, Aanensen DM, Harris SR 2018 Phandango: an interactive viewer for bacterial population genomics. Bioinformatics 34:292–293. doi:10.1093/bioinformatics/btx610. 29028899 PMC5860215

[57] Saber MM, Shapiro BJ 2020 Benchmarking bacterial genome-wide association study methods using simulated genomes and phenotypes. Microb Genom 6:e000337. doi:10.1099/mgen.0.000337. PMC7200059 32100713

[58] Wheeler NE, Reuter S, Chewapreecha C, Lees JA, Blane B, Horner C, Enoch D, Brown N, Estée Török M, Aanensen DM, Parkhill J, Peacock SJ 4 9 2019 Contrasting approaches to genome-wide association studies impact the detection of resistance mechanisms in *Staphylococcus aureus*. bioRxiv doi:10.1101/758144.

[59] Mignan A, Broccardo M 2019 One neuron versus deep learning in aftershock prediction. Nature 574:E1–E3. doi:10.1038/s41586-019-1582-8. 31578475

[60] Quinn TP, Erb I 29 11 2019 Another look at microbe–metabolite interactions: how scale invariant correlations can outperform a neural network. bioRxiv doi:10.1101/847475.

[61] Demczuk W, Lynch T, Martin I, Van Domselaar G, Graham M, Bharat A, Allen V, Hoang L, Lefebvre B, Tyrrell G, Horsman G, Haldane D, Garceau R, Wylie J, Wong T, Mulvey MR 2015 Whole-genome phylogenomic heterogeneity of *Neisseria gonorrhoeae* isolates with decreased cephalosporin susceptibility collected in Canada between 1989 and 2013. J Clin Microbiol 53:191–200. doi:10.1128/JCM.02589-14. 25378573 PMC4290921

[62] Bolger AM, Lohse M, Usadel B 2014 Trimmomatic: a flexible trimmer for Illumina sequence data. Bioinformatics 30:2114–2120. doi:10.1093/bioinformatics/btu170. 24695404 PMC4103590

[63] Bankevich A, Nurk S, Antipov D, Gurevich A. a, Dvorkin M, Kulikov AS, Lesin VM, Nikolenko SI, Pham S, Prjibelski AD, Pyshkin AV, Sirotkin AV, Vyahhi N, Tesler G, Alekseyev MA, Pevzner PA 2012 SPAdes: a new genome assembly algorithm and its applications to single-cell sequencing. J Comput Biol 19:455–477. doi:10.1089/cmb.2012.0021. 22506599 PMC3342519

[64] Lees JA, Harris SR, Tonkin-Hill G, Gladstone RA, Lo SW, Weiser JN, Corander J, Bentley SD, Croucher NJ 2019 Fast and flexible bacterial genomic epidemiology with PopPUNK. Genome Res 29:304–316. doi:10.1101/gr.241455.118. 30679308 PMC6360808

[65] Friedman J, Hastie T, Tibshirani R 2010 Regularization paths for generalized linear models via coordinate descent. J Stat Softw 33:1–22. 20808728 PMC2929880

[66] Chen PE, Shapiro BJ 2015 The advent of genome-wide association studies for bacteria. Curr Opin Microbiol 25:17–24. doi:10.1016/j.mib.2015.03.002. 25835153

[67] Young BC, Earle SG, Soeng S, Sar P, Kumar V, Hor S, Sar V, Bousfield R, Sanderson ND, Barker L, Stoesser N, Emary KR, Parry CM, Nickerson EK, Turner P, Bowden R, Crook DW, Wyllie DH, Day NP, Wilson DJ, Moore CE 2019 Panton-Valentine leucocidin is the key determinant of *Staphylococcus aureus* pyomyositis in a bacterial GWAS. Elife 8:e42486. doi:10.7554/eLife.42486. 30794157 PMC6457891

[68] Zhang C-H, Huang J 2008 The sparsity and bias of the lasso selection in high-dimensional linear regression. Ann Statist 36:1567–1594. doi:10.1214/07-AOS520.

[69] Galardini M, Clermont O, Baron A, Busby B, Dion S, Schubert S, Beltrao P, Denamur E 23 7 2019 Major role of the high-pathogenicity island (HPI) in the intrinsic extra-intestinal virulence of *Escherichia coli* revealed by a genome-wide association study. bioRxiv doi:10.1101/712034. PMC7592755 33112851

[70] Corander J, Fraser C, Gutmann MU, Arnold B, Hanage WP, Bentley SD, Lipsitch M, Croucher NJ 2017 Frequency-dependent selection in vaccine-associated pneumococcal population dynamics. Nat Ecol Evol 1:1950–1960. doi:10.1038/s41559-017-0337-x. 29038424 PMC5708525

[71] McNally A, Kallonen T, Connor C, Abudahab K, Aanensen DM, Horner C, Peacock SJ, Parkhill J, Croucher NJ, Corander J 2019 Diversification of colonization factors in a multidrug-resistant *Escherichia coli* lineage evolving under negative frequency-dependent selection. mBio 10:e00644-19. doi:10.1128/mBio.00644-19. 31015329 PMC6479005

[72] Fan J, Lv J 2008 Sure independence screening for ultrahigh dimensional feature space. J R Stat Soc Series B Stat Methodol 70:849–911. doi:10.1111/j.1467-9868.2008.00674.x. PMC2709408 19603084

[73] Carlsen M, Fu G, Bushman S, Corcoran C 2016 Exploiting linkage disequilibrium for ultrahigh-dimensional genome-wide data with an integrated statistical approach. Genetics 202:411–426. doi:10.1534/genetics.115.179507. 26661113 PMC4788225

[74] Drezen E, Rizk G, Chikhi R, Deltel C, Lemaitre C, Peterlongo P, Lavenier D 2014 GATB: Genome Assembly & Analysis Tool Box. Bioinformatics 30:2959–2961. doi:10.1093/bioinformatics/btu406. 24990603 PMC4184257

[75] Morcos F, Pagnani A, Lunt B, Bertolino A, Marks DS, Sander C, Zecchina R, Onuchic JN, Hwa T, Weigt M 2011 Direct-coupling analysis of residue coevolution captures native contacts across many protein families. Proc Natl Acad Sci U S A 108:E1293–E1301. doi:10.1073/pnas.1111471108. 22106262 PMC3241805

[76] Ferragina P, Manzini G 2005 Indexing compressed text. JACM 52:552–581. doi:10.1145/1082036.1082039.

[77] Döring A, Weese D, Rausch T, Reinert K 2008 SeqAn an efficient, generic C++ library for sequence analysis. BMC Bioinformatics 9:11. doi:10.1186/1471-2105-9-11. 18184432 PMC2246154

[78] Holley G, Melsted P 13 8 2019 Bifrost – highly parallel construction and indexing of colored and compacted de Bruijn graphs. bioRxiv doi:10.1101/695338. PMC7499882 32943081

[79] Lees JA, Galardini M, Bentley SD, Weiser JN, Corander J 2018 pyseer: a comprehensive tool for microbial pangenome-wide association studies. Bioinformatics 34:4310–4312. doi:10.1093/bioinformatics/bty539. 30535304 PMC6289128

[80] Croucher NJ, Walker D, Romero P, Lennard N, Paterson GK, Bason NC, Mitchell AM, Quail M. a, Andrew PW, Parkhill J, Bentley SP, Mitchell TJ 2009 Role of conjugative elements in the evolution of the multidrug-resistant pandemic clone *Streptococcus pneumoniae* Spain23F ST81. J Bacteriol 191:1480–1489. doi:10.1128/JB.01343-08. 19114491 PMC2648205

[81] Dempsey JA, Litaker W, Madhure A, Snodgrass TL, Cannon JG 1991 Physical map of the chromosome of *Neisseria gonorrhoeae* FA1090 with locations of genetic markers, including *opa* and *pil* genes. J Bacteriol 173:5476–5486. doi:10.1128/jb.173.17.5476-5486.1991. 1679431 PMC208260

[82] Schweiger R, Fisher E, Rahmani E, Shenhav L, Rosset S, Halperin E 2018 Using stochastic approximation techniques to efficiently construct confidence intervals for heritability. J Comput Biol 25:794–808. doi:10.1089/cmb.2018.0047. 29932739

[83] Grad YH, Harris SR, Kirkcaldy RD, Green AG, Marks DS, Bentley SD, Trees D, Lipsitch M 2016 Genomic epidemiology of gonococcal resistance to extended-spectrum cephalosporins, macrolides, and fluoroquinolones in the United States, 2000-2013. J Infect Dis 214:1579–1587. doi:10.1093/infdis/jiw420. 27638945 PMC5091375

[84] De Silva D, Peters J, Cole K, Cole MJ, Cresswell F, Dean G, Dave J, Thomas DR, Foster K, Waldram A, Wilson DJ, Didelot X, Grad YH, Crook DW, Peto TE, Walker AS, Paul J, Eyre DW 2016 Whole-genome sequencing to determine transmission of *Neisseria gonorrhoeae*: an observational study. Lancet Infect Dis 16:1295–1303. doi:10.1016/S1473-3099(16)30157-8. 27427203 PMC5086424

[85] Croucher NJ, Finkelstein JA, Pelton SI, Parkhill J, Bentley SD, Lipsitch M, Hanage WP 2015 Population genomic datasets describing the post-vaccine evolutionary epidemiology of *Streptococcus pneumoniae*. Sci Data 2:150058. doi:10.1038/sdata.2015.58. 26528397 PMC4622223

