# Supplementary material for: Improved Prediction of Bacterial Genotype-Phenotype Associations Using Interpretable Pangenome-Spanning Regressions
Source: mBio. 2020 Jul 7;11(4):e01344-20. doi: 10.1128/mBio.01344-20 (PMC7343994; doi:10.1128/mBio.01344-20)
Supplement: TABLE S1 [file mBio.01344-20-st001.pdf]

Drug	Model	Variants Selected	Lineage	R^2	TP	TN	FP	FN
Rifampicin	Weighted	90	All	0.84	752	388	32	11
			1	0.42	63	354	26	9
			2	0.76	420	22	4	2
			3	0.83	201	5	1	0
			4	0.79	68	4	1	0
	Unweighted	132	All	0.86	755	388	29	8
			1	0.42	65	356	24	7
			2	0.76	421	23	3	1
			3	0.83	201	5	1	0
			4	0.79	68	4	1	0
	WDNN		All	0.84	723	411	9	35
Isoniazid	Weighted	151	All	0.85	657	487	38	6
			1	0.52	43	394	18	1
			2	0.69	389	59	14	5
			3	0.80	166	19	4	0
			4	0.85	59	15	2	0
	Unweighted	185	All	0.84	657	484	41	6
			1	0.45	43	391	21	1
			2	0.69	389	59	14	5
			3	0.80	166	19	4	0
			4	0.85	59	15	2	0
	WDNN		All	0.72	599	506	19	64
Ethambutol	Weighted	32	All	0.59	799	278	57	41
			1	0.13	89	272	39	41
			2	0.25	415	6	15	0
			3	-	209	0	0	0
			4	-	86	0	3	0
	Unweighted	258	All	0.65	803	289	46	37
			1	0.27	97	277	34	33
			2	0.25	411	10	11	4
			3	-	209	0	0	0
			4	0.67	86	2	1	0
	WDNN		All	0.47	761	287	48	79
Pyrazinamide	Weighted	192	All	0.61	775	202	39	33
			1	0.28	82	189	21	32
			2	0.34	413	10	14	1
			3	-	195	0	2	0
			4	0.58	85	3	2	0
	Unweighted	328	All	0.66	781	204	37	27
			1	0.36	90	187	23	24
			2	0.43	411	14	10	3
			3	-	195	0	2	0
			4	0.58	85	3	2	0
	WDNN		All	-0.19	608	220	21	200
