# Supplementary figures and images for: Improved Prediction of Bacterial Genotype-Phenotype Associations Using Interpretable Pangenome-Spanning Regressions
Source: mBio. 2020 Jul 7;11(4):e01344-20. doi: 10.1128/mBio.01344-20 (PMC7343994; doi:10.1128/mBio.01344-20)

SPARC

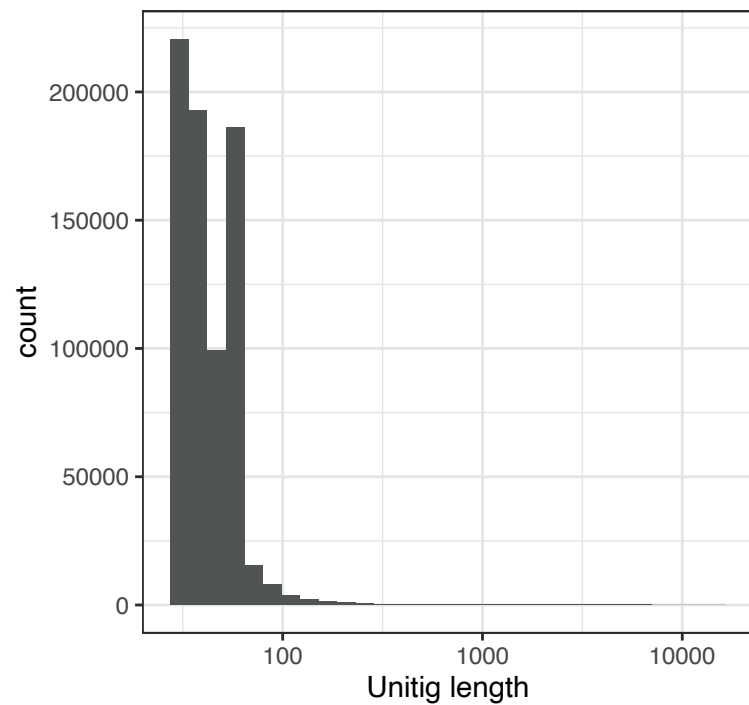

Supplement: FIG S1 [file mBio.01344-20-sf001.pdf]

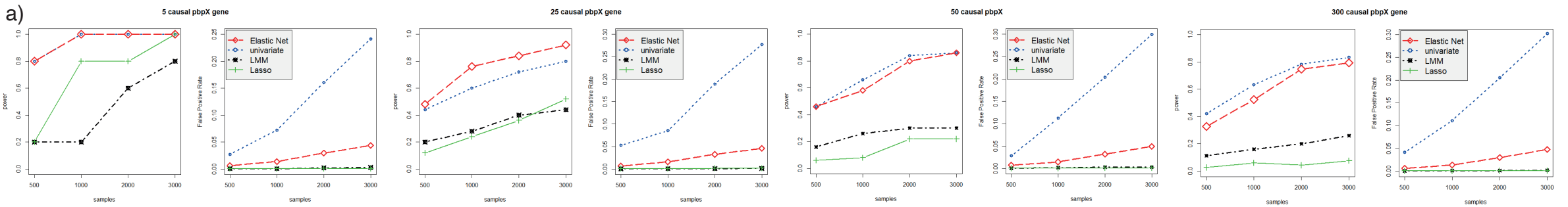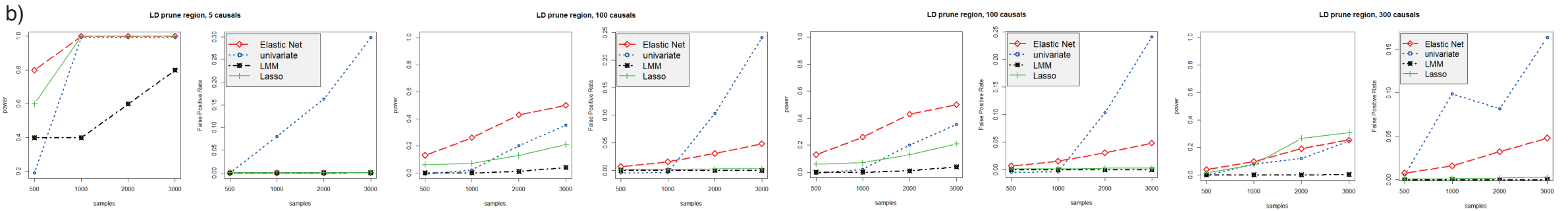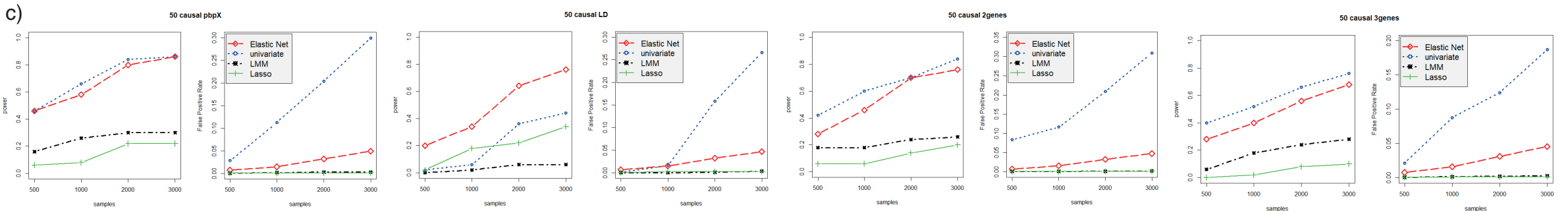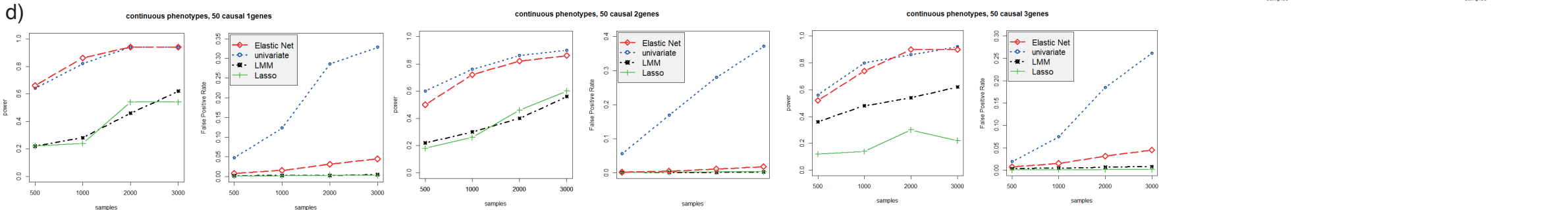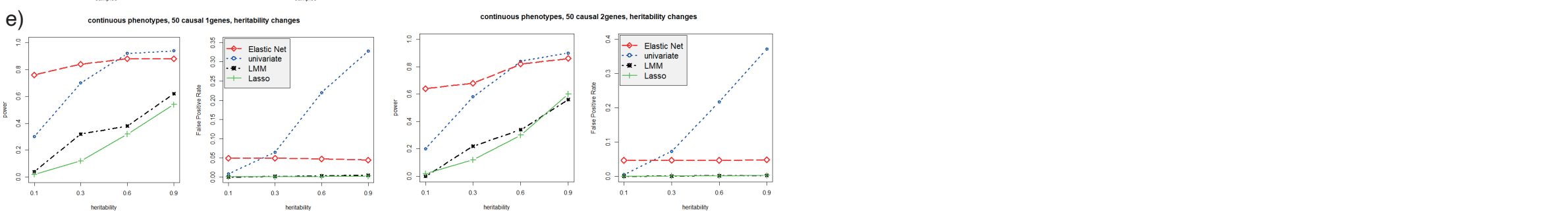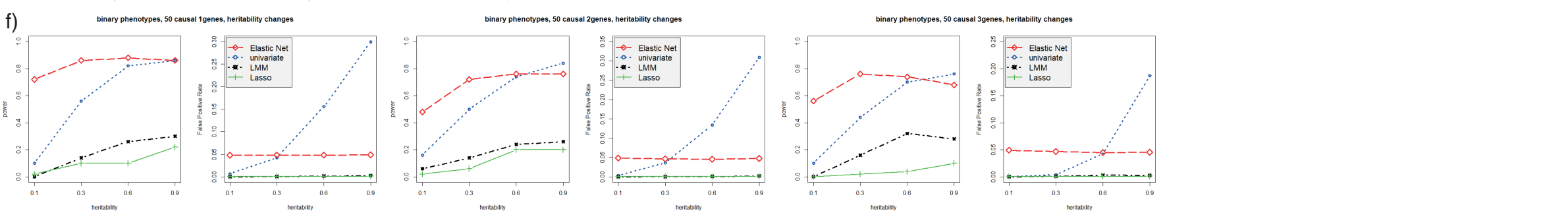

Supplement: FIG S2 [file mBio.01344-20-sf002.pdf]

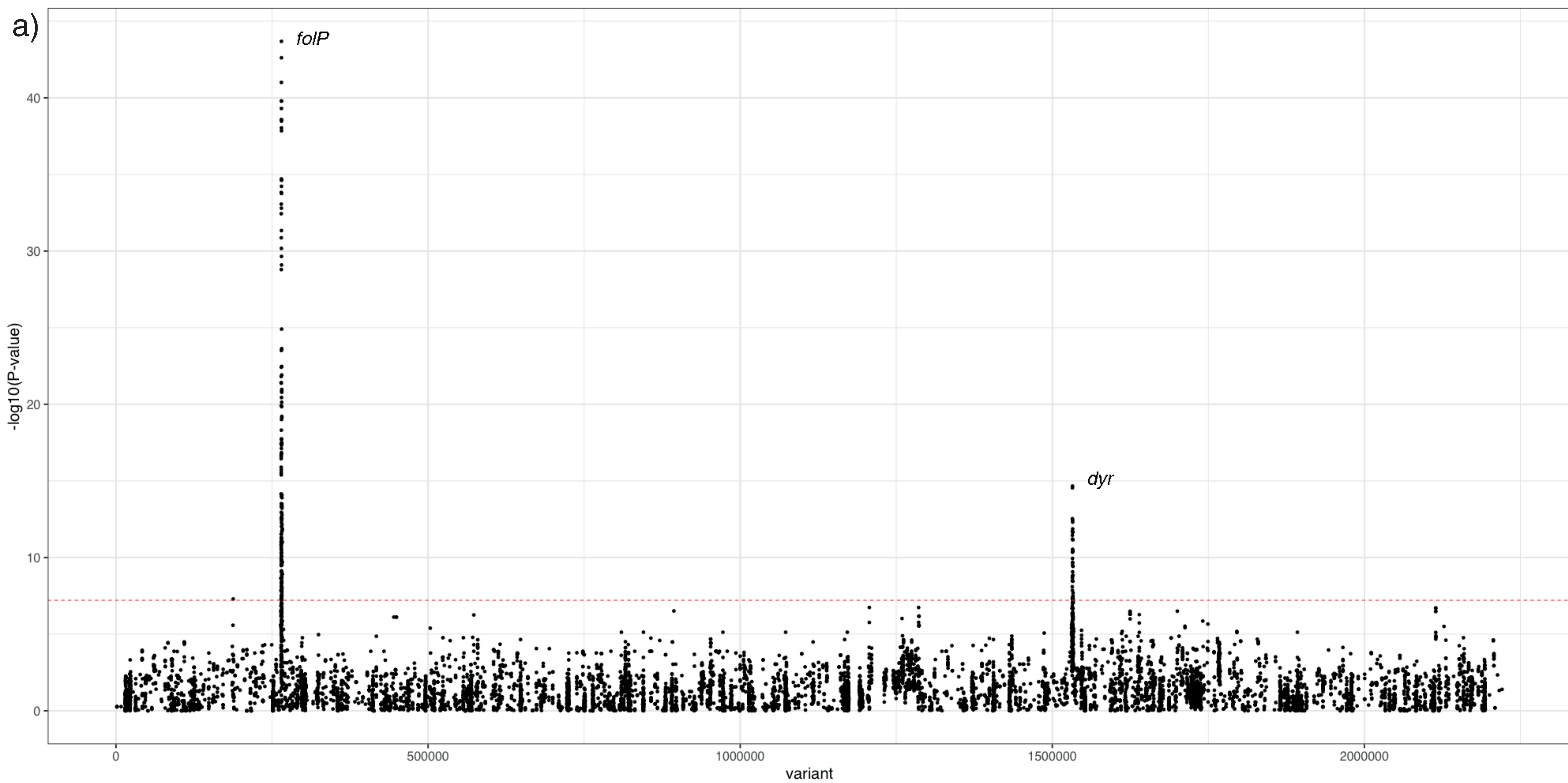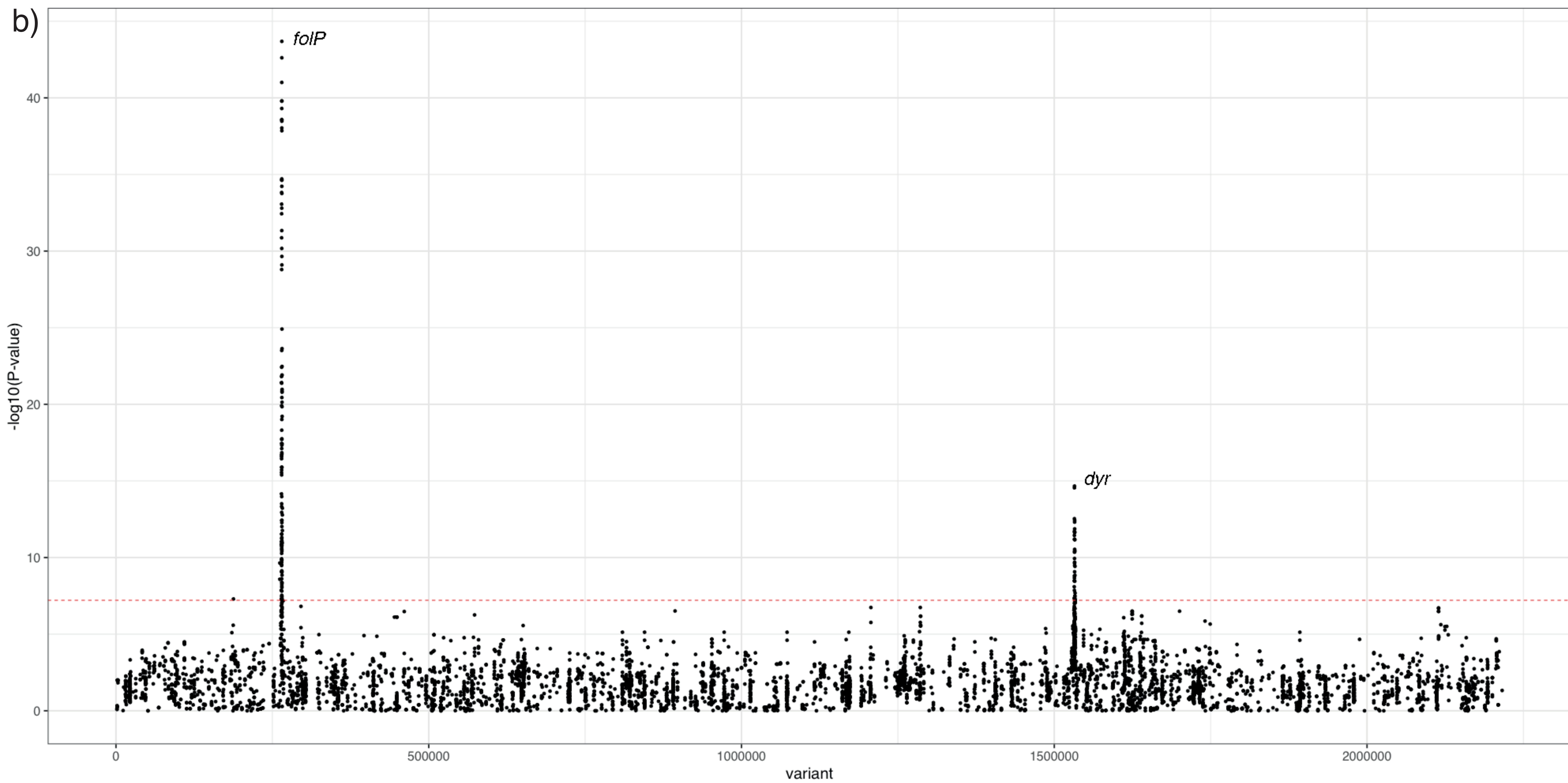

Supplement: FIG S3 [file mBio.01344-20-sf003.pdf]

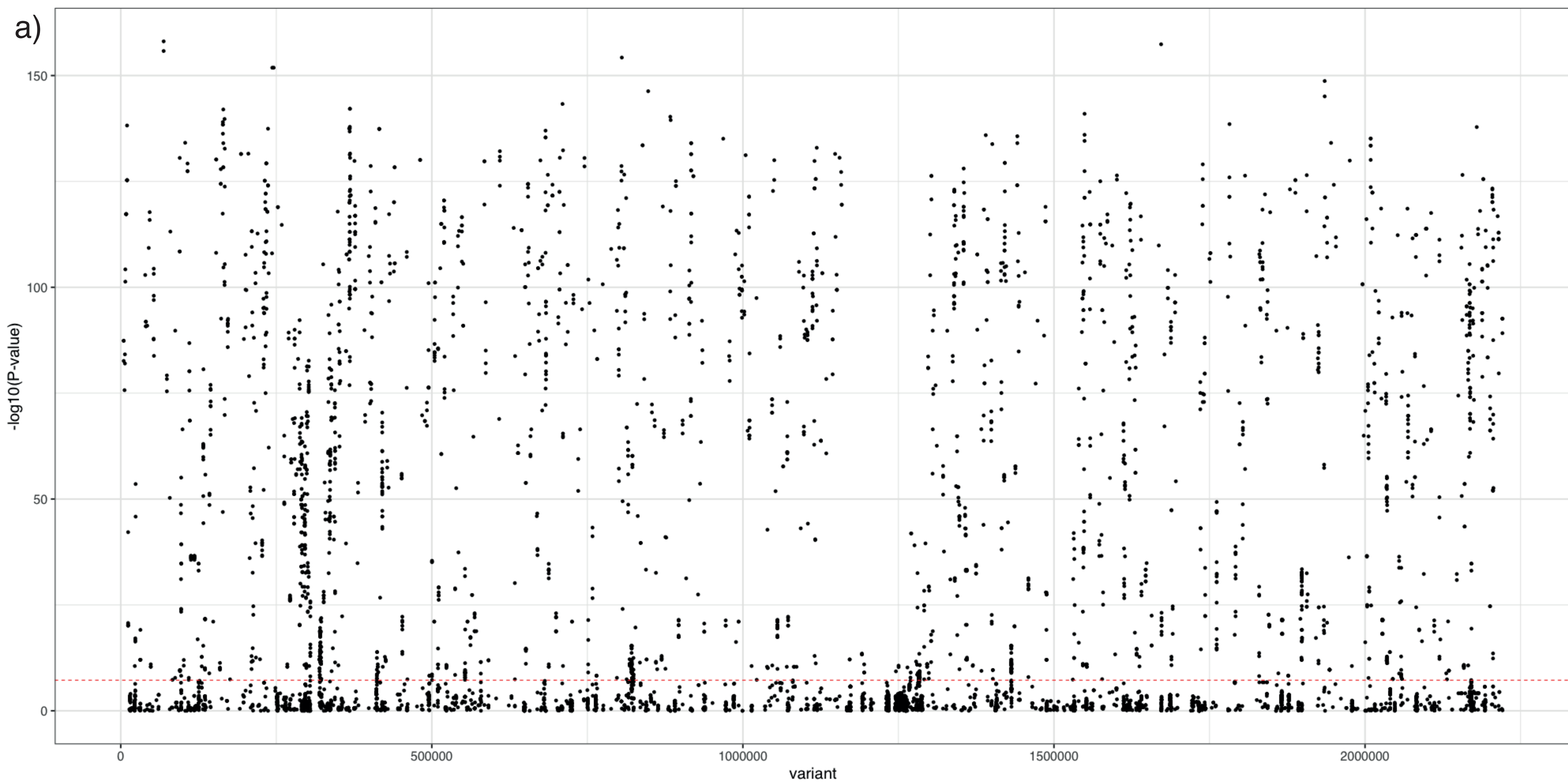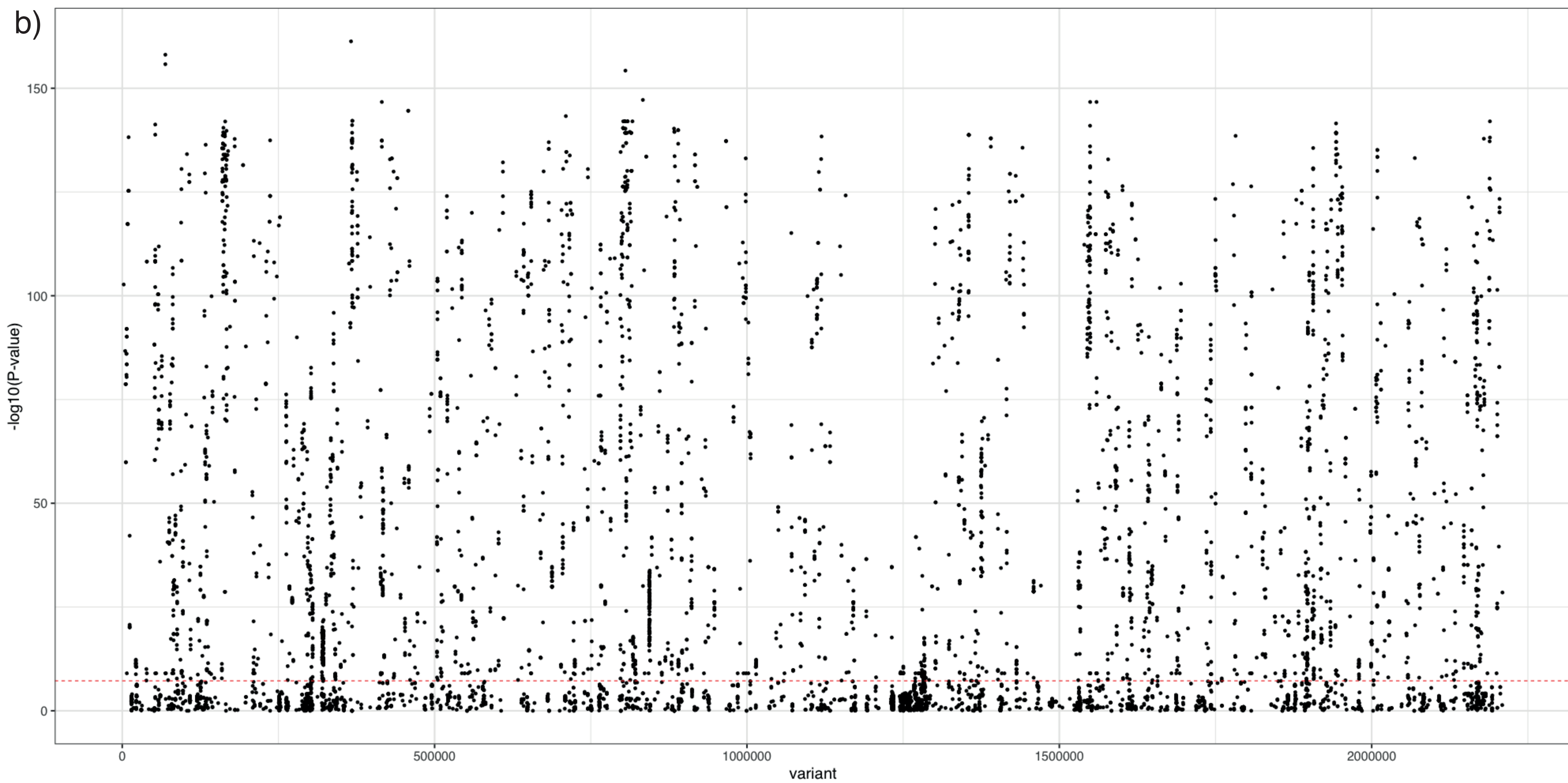

Supplement: FIG S4 [file mBio.01344-20-sf004.pdf]

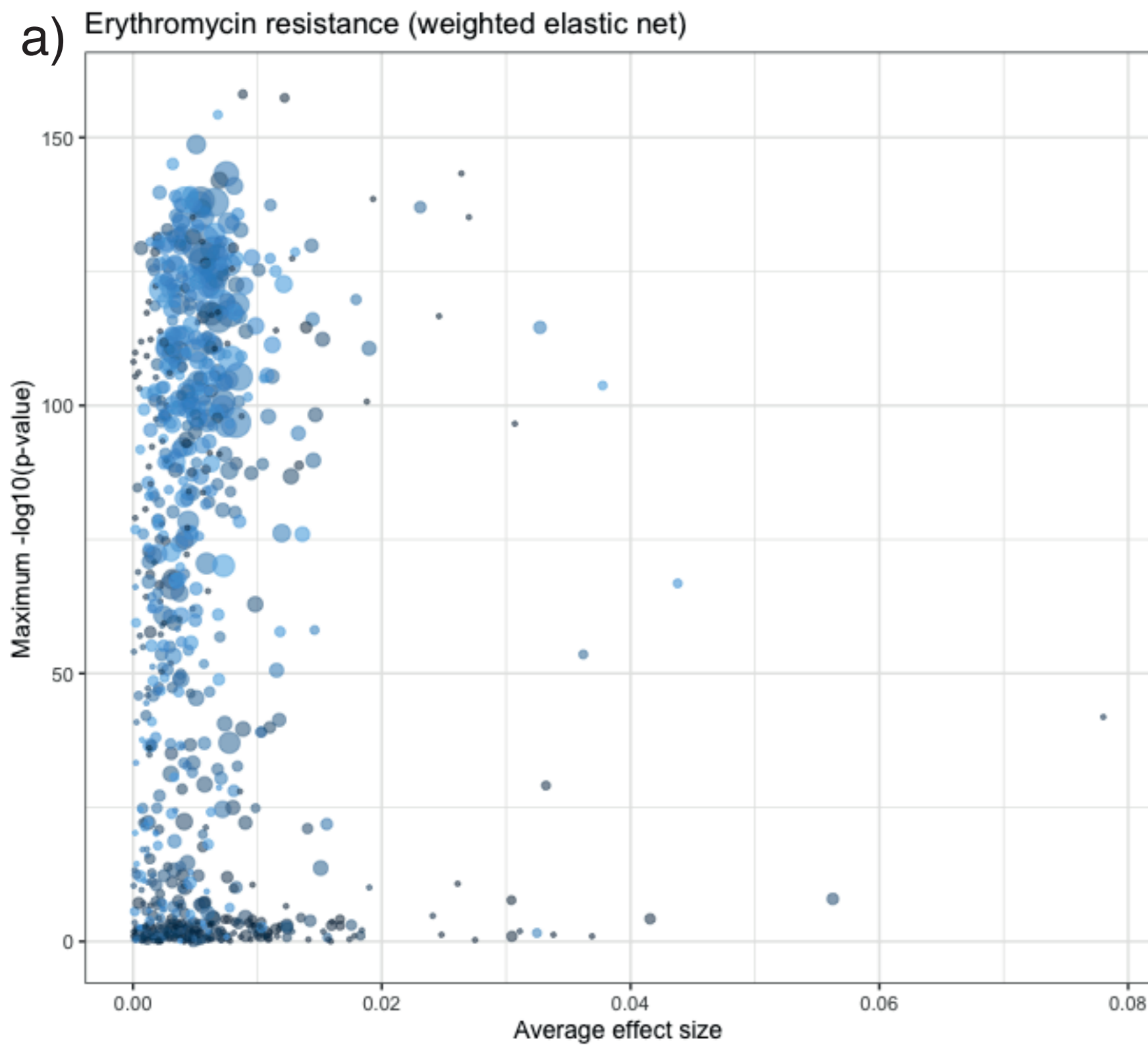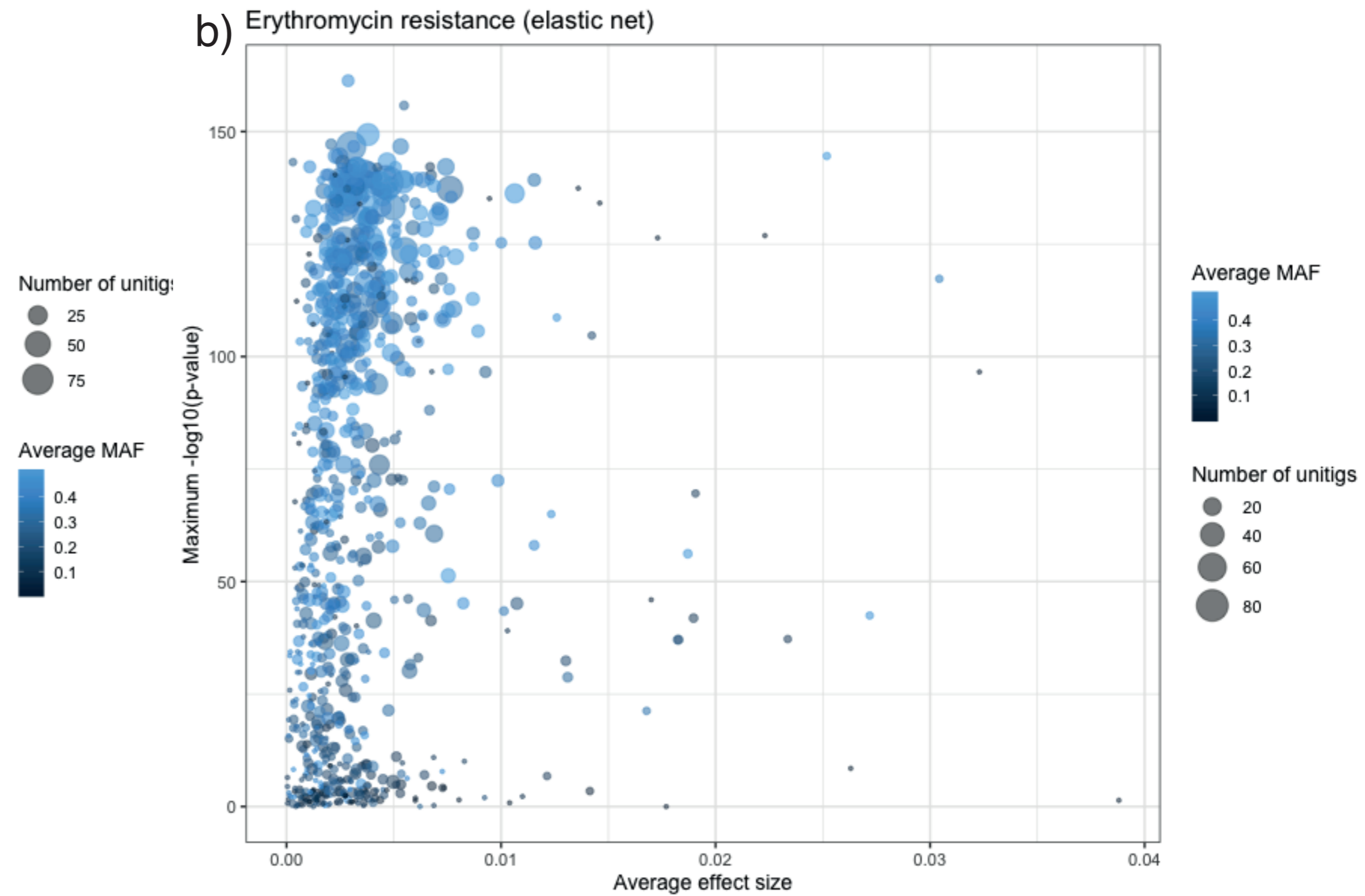

Supplement: FIG S5 [file mBio.01344-20-sf005.pdf]

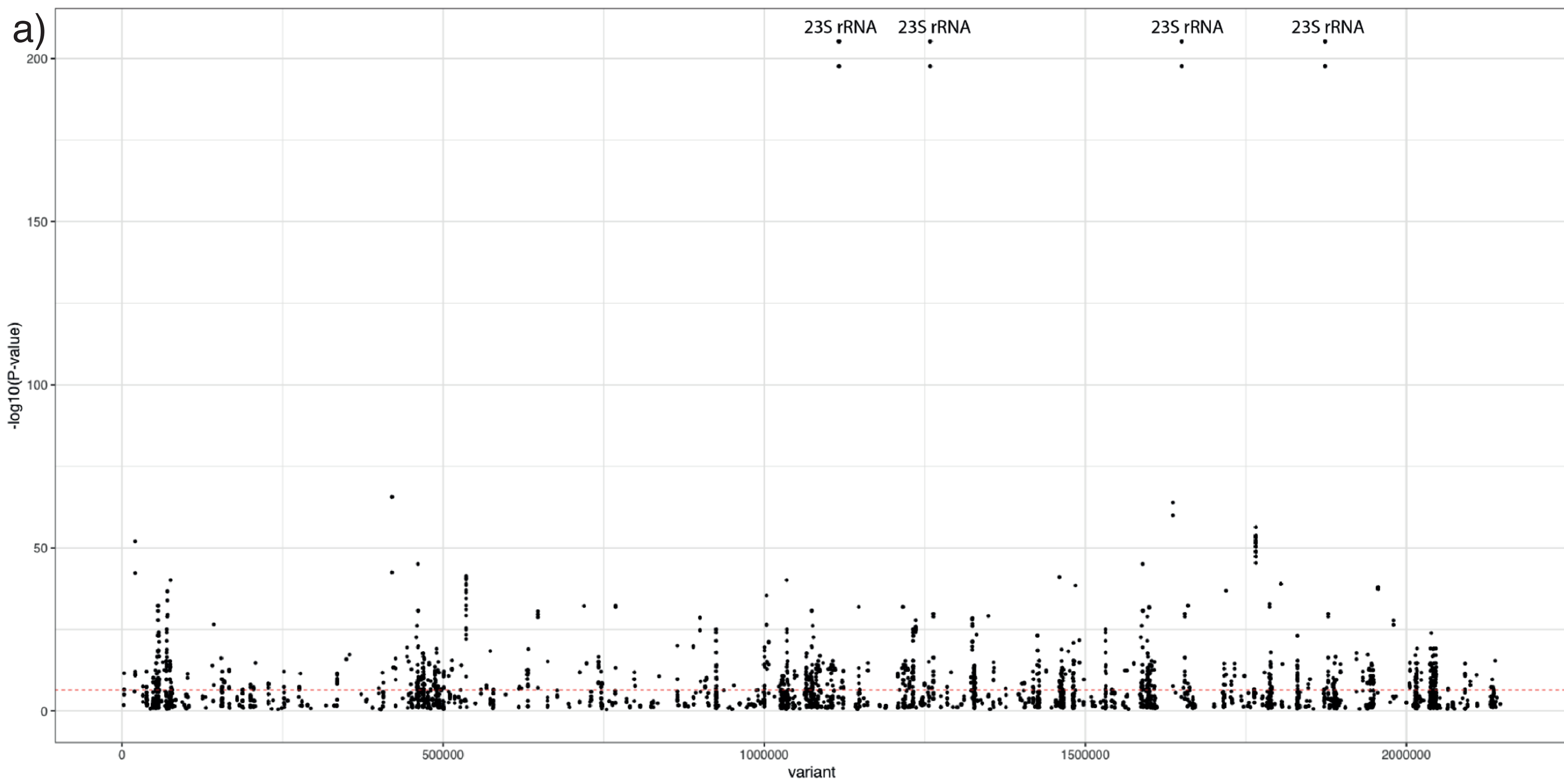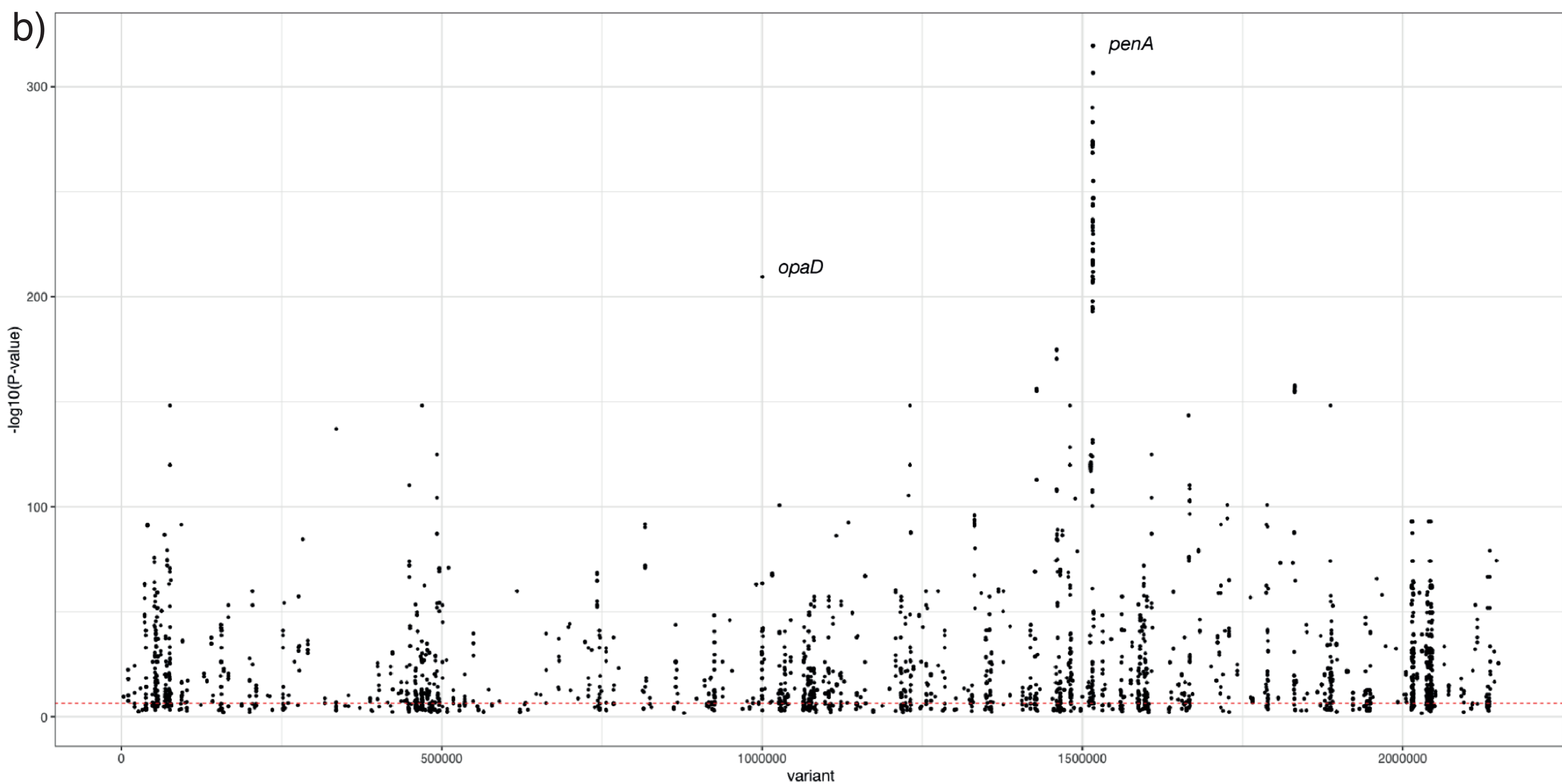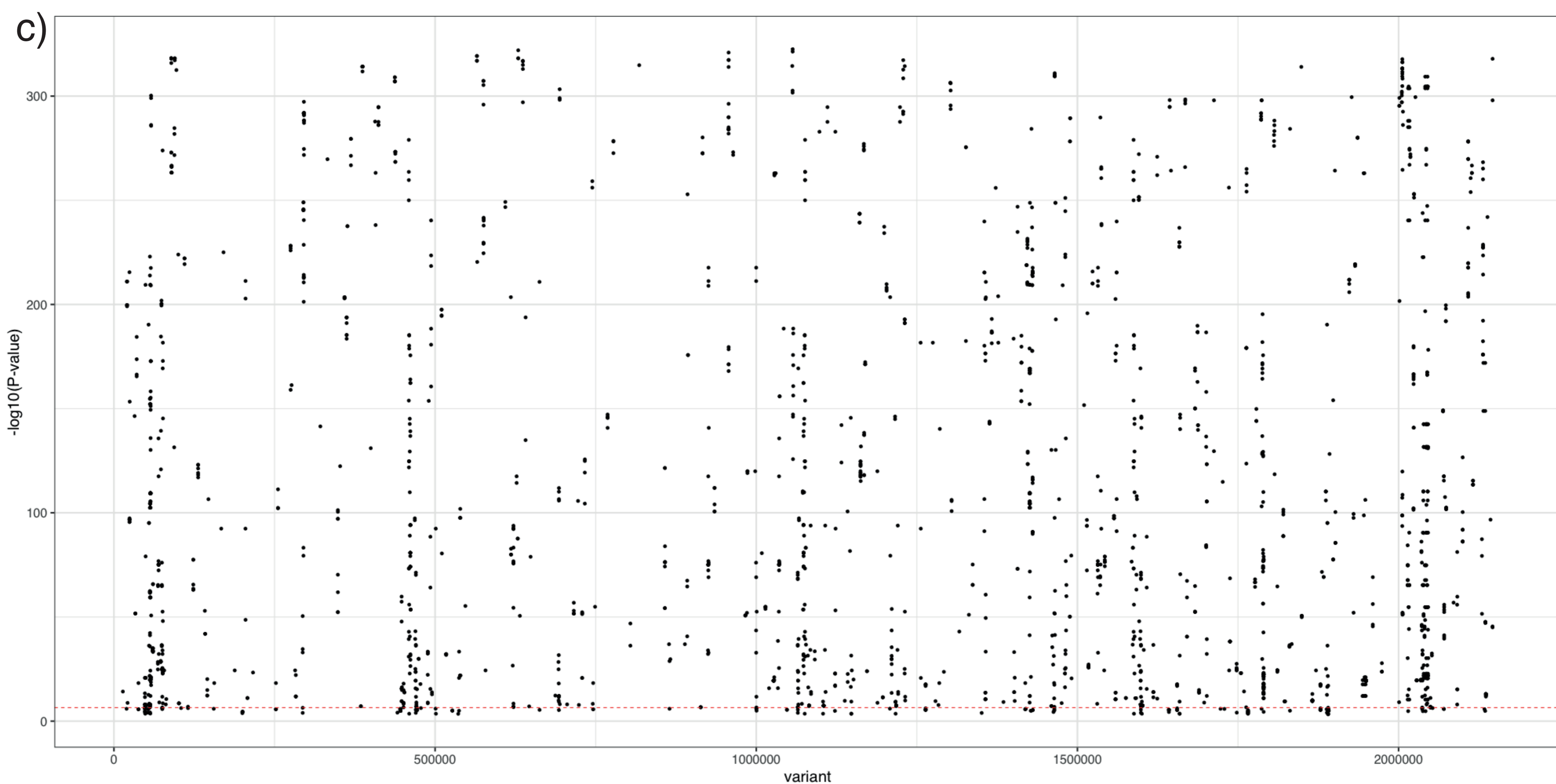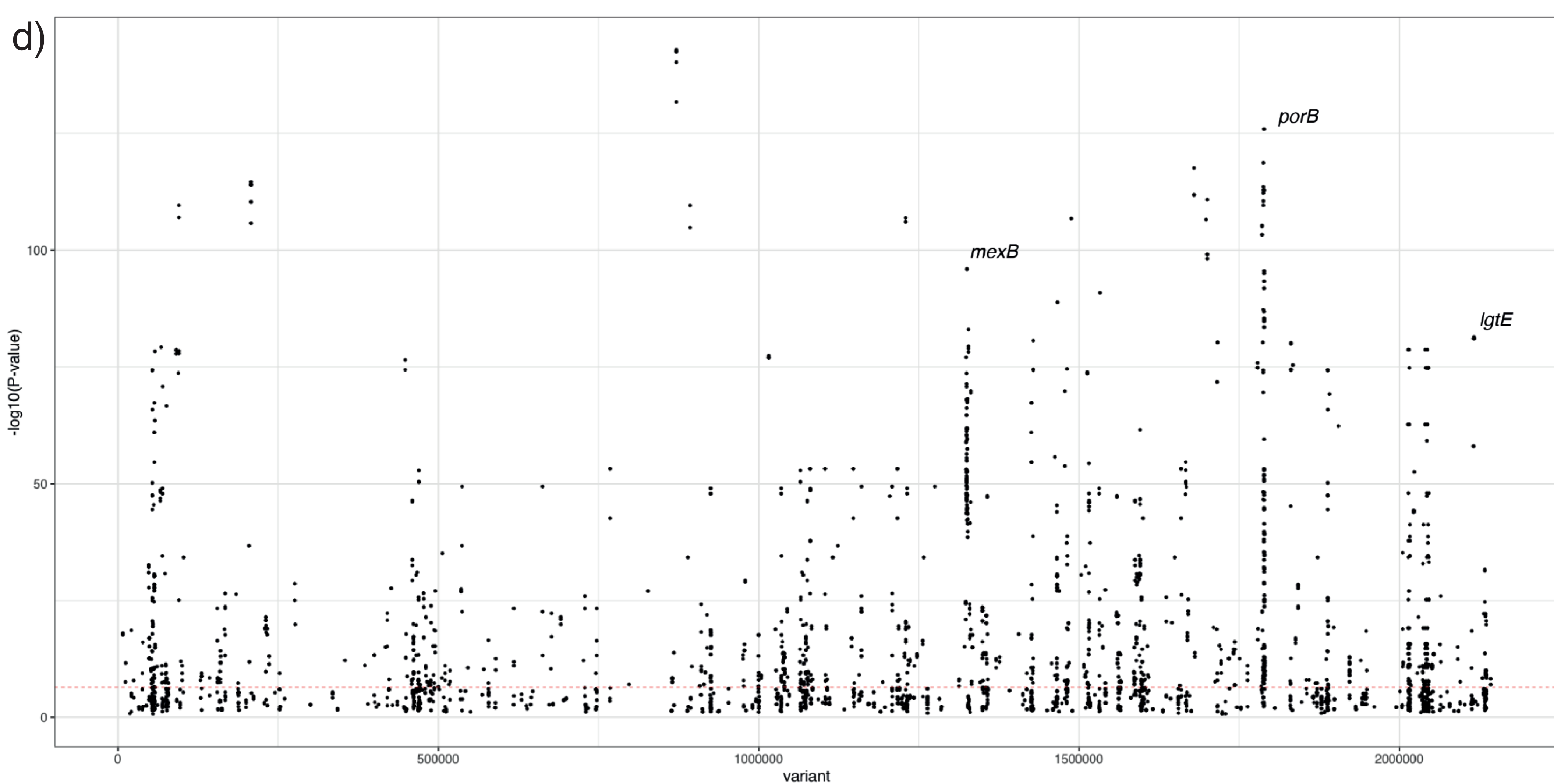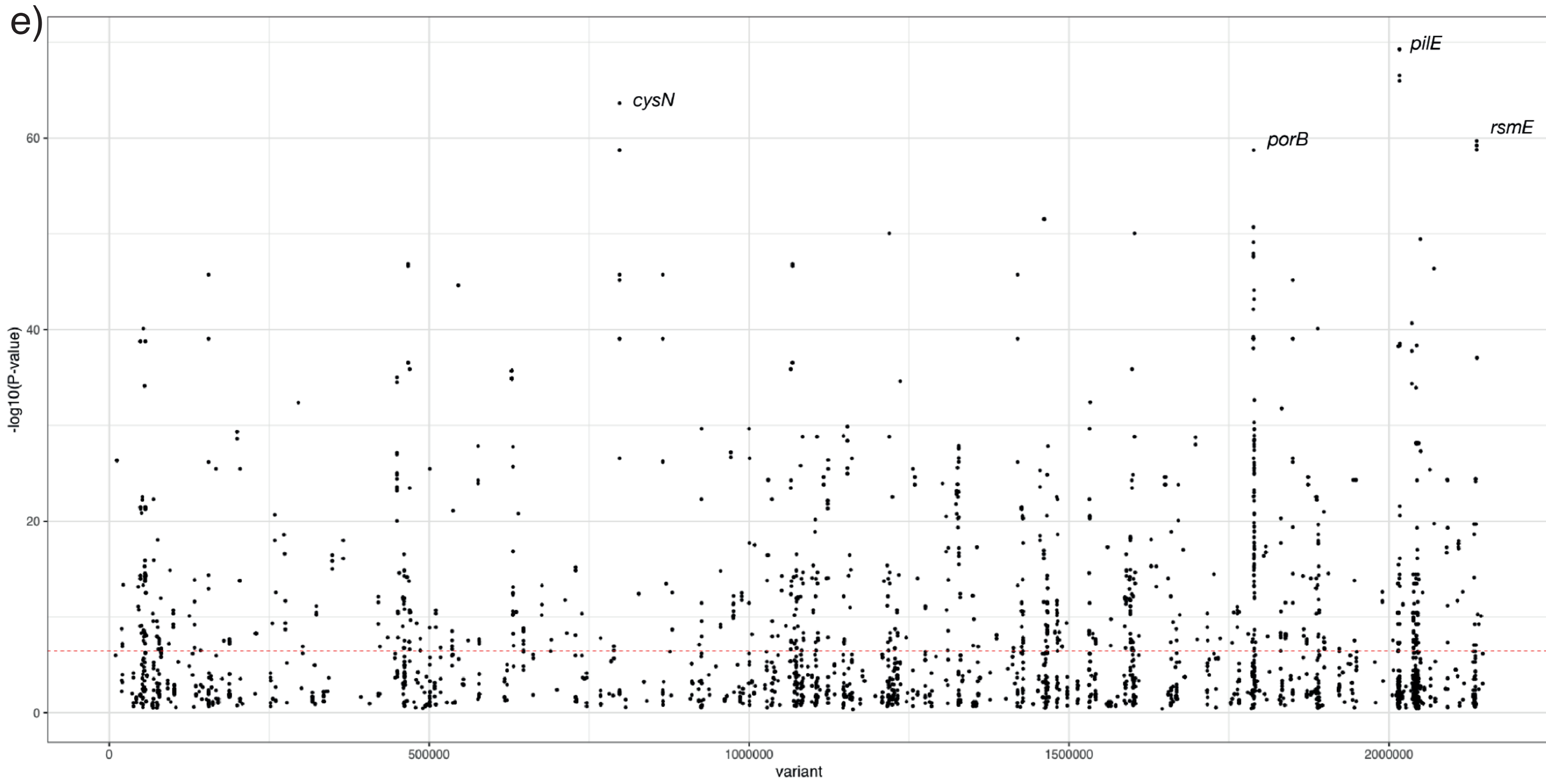

Supplement: FIG S6 [file mBio.01344-20-sf006.pdf]
