# Supplementary material for: Improved Prediction of Bacterial Genotype-Phenotype Associations Using Interpretable Pangenome-Spanning Regressions
Source: mBio. 2020 Jul 7;11(4):e01344-20. doi: 10.1128/mBio.01344-20 (PMC7343994; doi:10.1128/mBio.01344-20)
Supplement: TABLE S2 [file mBio.01344-20-st002.pdf]

Dataset	Phenotype	R^2	TP	TN	FP	FN
GAS	Virulence	-0.05	327	107	752	34
SPARC	Penicillin	0.81	71	121	63	5
	Erythromycin	0.61	11	161	15	17
Netherlands	Meningitis	0.65	354	208	22	28
