# Supplementary material for: Improved Prediction of Bacterial Genotype-Phenotype Associations Using Interpretable Pangenome-Spanning Regressions
Source: mBio. 2020 Jul 7;11(4):e01344-20. doi: 10.1128/mBio.01344-20 (PMC7343994; doi:10.1128/mBio.01344-20)
Supplement: TABLE S3 [file mBio.01344-20-st003.pdf]

	thresholds	500	1000	2000	3000
5 snps	the 1st Qu.	5 (90248)	5 (90746)	5 (90658)	5 (90742)
	the median.	5 (60411)	5 (60506)	5 (60507)	5 (60507)
	the 3rd Qu.	4 (30247)	5 (30252)	4 (30254)	5 (30254)
25 snps	the 1st Qu.	25 (90690)	25 (90425)	25 (90721)	25 (90758)
	the median.	23 (60216)	20 (60506)	23 (60507)	22 (60501)
	the 3rd Qu.	14 (30222)	15 (30254)	15 (30254)	15 (30254)
100 snps	the 1st Qu.	94 (90273)	95 (90733)	94 (90739)	95 (90729)
	the median.	74 (57564)	77 (60487)	79 (60506)	74 (60427)
	the 3rd Qu.	49 (30225)	50 (30254)	51 (30254)	48 (30254)
300 snps	the 1st Qu.	273 (87892)	284 (90661)	283 (90681)	277 (90760)
	the median	219 (60479)	219 (60501)	219 (60506)	217 (60494)
	the 3rd Qu.	148 (30253)	145 (30250)	141 (30254)	144 (30254)

	threshold	500	1000	2000	3000
5 snps	the 1st Qu.	5 (90501)	5 (90755)	5 (90760)	5 (90759)
	the median	5 (60343)	5 (60506)	5 (60495)	5 (60505)
	the 3rd Qu.	3 (30239)	3 (30245)	3 (30254)	3 (30254)
25 snps	the 1st Qu.	25 (89728)	25 (90755)	25 (90743)	25 (90759)
	the median	22 (60046)	22 (60410)	22 (60501)	22 (60485)
	the 3rd Qu.	12 (30253)	13 (30254)	13 (30254)	12 (30254)
100 snps	the 1st Qu.	96 (90063)	96 (90757)	97 (90671)	97 (90752)
	the median.	86 (60489)	88 (60458)	94 (60507)	90 (60505)
	the 3rd Qu.	56 (30254)	55 (30240)	57 (30263)	57 (30254)
300 snps	the 1st Qu.	284 (90632)	291 (90760)	289 (90742)	289 (90760)
	the median	233 (60497)	254 (60497)	268 (60506)	264 (60507)
	the 3rd Qu.	165 (30250)	168 (30252)	164 (30252)	166 (60254)

	threshold	500	1000	2000	3000
50 snps (LD-prune)	the 1st Qu.	49 (90488)	50 (90595)	50 (90757)	50 (90737)
	the median	43 (60450)	45 (60438)	45 (60479)	41 (60491)
	the 3rd Qu.	32 (30254)	33 (30251)	33 (30253)	33 (30254)
50 snps (gene *pbpX*)	the 1st Qu.	49 (90589)	49 (90753)	49 (90759)	49 (90756)
	the median	43 (60054)	45 (60501)	46 (60507)	45 (60465)
	the 3rd Qu.	34 (30251)	32 (30243)	33 (30254)	32 (30253)
50 snps (50% gene *pbpX*, 50% gene *penA*)	the 1st Qu.	47 (90733)	48 (90753)	48 (90754)	48 (90750)
	the median.	40 (60495)	44 (60123)	43 (60506)	42 (60490)
	the 3rd Qu.	25 (30158)	27 (30253)	27 (30254)	26 (30254)
50 snps (16 from gene *pbpX*, 17 from gene *penA*, 17 from gene *penX*)	the 1st Qu.	49 (90720)	49 (90759)	49 (90752)	49 (90757)
	the median	46 (59328)	47 (60459)	46 (60471)	46 (60499)
	the 3rd Qu.	34 (30232)	34 (30254)	35 (30254)	35 (30254)

	threshold	$h^2 = 0.1$	$h^2 = 0.3$	$h^2 = 0.6$	$h^2 = 0.9$
50 snps (gene *pbpX*)	the 1st Qu.	44 (90760)	44 (90760)	45 (90760)	44 (90760)
	the median	44 (60507)	44 (60507)	44 (60507)	44 (60507)
	the 3rd Qu.	42 (29977)	41 (30234)	42 (30180)	43 (30254)
50 snps (50% gene *pbpX*, 50% gene *penA*)	the 1st Qu.	44 (90760)	45 (90760)	45 (90760)	45 (90760)
	the median.	43 (60507)	43 (60507)	43 (60494)	43 (60507)
	the 3rd Qu.	42 (30254)	41 (30254)	41 (30254)	41 (30254)
50 snps (16 from gene *pbpX*, 17 from gene *penA*, 17 from gene *penX*)	the 1st Qu.	49 (90760)	48 (90760)	48 (90760)	48 (60760)
	the median	48 (60507)	47 (60507)	47 (60507)	47 (60506)
	the 3rd Qu.	41 (30254)	42 (30253)	42 (30254)	43 (30254)
